# Multicentric origin and diversification of *atp6*‐*orf79*‐like structures reveal mitochondrial gene flows in *Oryza rufipogon* and *Oryza sativa*

**DOI:** 10.1111/eva.13022

**Published:** 2020-06-27

**Authors:** Wenchuang He, Caijin Chen, Yawo Mawunyo Nevame Adedze, Xilong Dong, Kun Xi, Yongsheng Sun, Tengfei Dang, Deming Jin

**Affiliations:** ^1^ MOA Key Laboratory of Crop Ecophysiology and Farming System in the Middle Reaches of the Yangtze River College of Plant Science and Technology Huazhong Agricultural University Wuhan China; ^2^ Institute of Biological and Environmental Sciences University of Aberdeen Aberdeen UK; ^3^ Molecular Biology Laboratory Jiangsu Green Port Modern Agriculture Development Company Suqian China

**Keywords:** biogeography, cytoplasmic male sterility, mitochondrial genome, *Oryza rufipogon*, *Oryza sativa*, phylogenetics

## Abstract

Cytoplasmic male sterility (CMS) is a widely used genetic tool in modern hybrid rice breeding. Most genes conferring rice gametophytic CMS are homologous to *orf79* and co‐transcribe with *atp6*. However, the origin, differentiation and flow of these mitochondrial genes in wild and cultivated rice species remain unclear. In this study, we performed de novo assembly of the mitochondrial genomes of 221 common wild rice (*Oryza rufipogon* Griff.) and 369 Asian cultivated rice (*Oryza sativa* L.) accessions, and identified 16 haplotypes of *atp6‐orf79*‐like structures and 11 *orf79* alleles. These homologous structures were classified into 4 distinct groups (AO‐I, AO‐II, AO‐III and AO‐IV), all of which were observed in *O. rufipogon* but only AO‐I was detected in *O. sativa*, causing a decrease in the frequency of *atp6‐orf79*‐like structures from 19.9% to 8.1%. Phylogenetic and biogeographic analyses revealed that the different groups of these gametophytic CMS‐related genes in *O. rufipogon* evolved in a multicentric pattern. The geographical origin of the *atp6‐orf79*‐like structures was further traced back, and a candidate region in north‐east of Gangetic Plain on the Indian Peninsula (South Asia) was identified as the origin centre of AO‐I. The *orf79* alleles were detected in all three cytoplasmic types (Or‐CT0, Or‐CT1 and Or‐CT2) of *O. rufipogon*, but only two alleles (*orf79a* and *orf79b*) were observed in Or‐CT0 type of *O. sativa*, while no *orf79* allele was found in other types of *O. sativa*. Our results also revealed that the *orf79* alleles in cultivated rice originated from the wild rice population in South and South‐East Asia. In addition, strong positive selection pressure was detected on the sequence variations of *orf79* alleles, and a special evolutionary strategy was noted in these gametophytic CMS‐related genes, suggesting that their divergence could be beneficial to their survival in evolution.

## 1 INTRODUCTION

Cytoplasmic male sterility (CMS) is a maternally inherited trait characterized by the disability to produce functional pollen; it has been observed in many species of higher plants and is widely used in commercial hybrid seed production. Molecular studies indicate that CMS is usually associated with chimeric open reading frames (ORFs) coding for abnormal (toxic) proteins in the mitochondrial genome (Bentolila, Alfonso, & Hanson, 2002), including homologous sequences corresponding to essential genes coding for ATPase, cytochrome c oxidase or ribosomal proteins (Arrieta‐Montiel & Mackenzie, 2011; Chen & Liu, 2014; Tang et al., 2017). These chimeric ORFs are typically thought to originate from illegitimate recombination events between normal mitochondrial genes and gene‐flanking sequences.

Rice CMS has been intensively studied and has been widely used in commercial production for decades. To date, more than 60 rice CMS lines have been developed by hybridization between species, subspecies and varieties; it can be mainly categorized into two major types, sporophytic and gametophytic CMS (Li, Yang, & Zhu, 2007). In these two types, the sterility of male gametes is dependent on the sporophyte and gametophyte genotypes, respectively. Three distinct sporophytic CMS genes have been cloned in rice, *WA352* and *WA314* in wild abortive‐type CMS (WA‐CMS) and *orf182* in D1‐CMS (Luo et al., 2013; Tang et al., 2017; Xie et al., 2018), although their cytoplasms are derived from the same wild rice species, *Oryza rufipogon*. Most gametophytic CMS‐related genes share homologous sequences of the same gene; for example, *orf79* (here denoted *orf79a*), *L‐orf79* (*orf79b*) and *orfH79* (*orf79k*) are considered as the functional genes of Boro II‐type CMS (BT‐CMS), Lead rice‐type CMS (LD‐CMS) and Hong‐Lian‐type CMS (HL‐CMS), respectively, but their cytoplasms are derived from Chinsurah Boro II (*Oryza sativa* ssp. *indica*), Burmese cultivar Lead rice (*O. sativa* ssp. *indica*) and red‐awned wild rice (*O. rufipogon*), respectively (Kazama, Itabashi, Fujii, Nakamura, & Toriyama, 2016; Peng et al., 2010; Shinjyo, 1969; Wang et al., 2006; Watanabe, 1971; Yingsheng, 1988). These *orf79* alleles are all co‐transcribed with the upstream *atp6* in the form of *atp6‐orf79*‐like structures; they can induce gametophyte abortion with different physiological mechanisms such as cytotoxicity caused by the accumulation of ORF79 and L‐ORF79 mainly in microspores, or energy deficiency resulting from the reduction of the enzymatic activity of mitochondrial complex III. The amount of ORF79 protein produced in LD‐CMS is considerably lower than that in BT‐CMS (Itabashi, Kazama, & Toriyama, 2009; Kazama et al., 2016). These *atp6‐orf79*‐like structures share high nucleotide sequence similarity and play a very important role in the molecular mechanism underlying gametophytic CMS, suggesting a possible origin from a common ancient genotype of *atp6‐orf79*.

Previous studies have also focused on the variations of the known gametophytic CMS genes and variant haplotypes of *orf79* (Duan, Li, Li, Xiong, & Zhu, 2007; Duan, Zheng, Yan, He, & Liao, 2015; Li, Tan, Wang, Wan, & Zhu, 2008; Luan et al., 2013). However, as the formation and evolution patterns of *WA352*‐like structures for sporophytic WA‐CMS have been elaborately studied (Tang et al., 2017), the origin and diversification of mitochondrial *atp6*‐*orf79*‐like structures for gametophytic CMS, such as phylogenetic and geographic relationships among them as well as how they originated and spread in the wild and cultivated rice species, remain obscure. Furthermore, massive and accurate assembly of mitochondrial genomes is difficult owing to the rapid variation in the noncoding region and complex rearrangement of mitochondrial genes (Knoop, 2004) and due to their exchange of fragments with nuclear and plastid genomes (Timmis, Ayliffe, Huang, & Martin, 2004). These difficulties limited the molecular characterization of mitochondrial genomes of plants at the population level; thus, only a limited number of complete mitochondrial genomes have been successfully assembled using whole genome sequencing (WGS) data set in plants (Donnelly et al., 2017; Iorizzo et al., 2012; Zimmermann et al., 2019) with the accumulation of high‐throughput sequencing data in recent years. However, the variation rates of the coding region of mitochondrial genes were found to be very low, even lower than those in nuclear and plastid genes (Muse, 2000; Wolfe, Li, & Sharp, 1987). Hence, mitochondrial protein‐coding genes could be readily assembled with WGS data sets in large scale.

In this study, a wide‐scope screening of homologous structures related to *atp6*‐*orf79* was attempted by assembling and assessing draft mitochondrial genomes of 590 common wild rice (*O. rufipogon*) and Asian cultivated rice (*O. sativa*) genotypes based on WGS data set in previous studies (Huang et al., 2012; Wang et al., 2018); and 16 *atp6*‐*orf79*‐like structures and 11 *orf79* alleles were obtained. Distinct groups and multicentric features were observed during the analyses of phylogenetic and biogeographic diversification, revealing different evolutionary routes of these gametophytic CMS‐related genes. Their geographical origin was deduced, and it showed a complex multi‐original process. Furthermore, novel evidences were provided to confirm the distinct and continuous mitochondrial gene flows during the diversification of common wild rice and domestication of Asian cultivated rice. In addition, a strong positive selection pressure was detected on sequence variations of the *orf79* alleles, indicating a special evolutionary strategy of these gametophytic CMS‐related genes, so that their divergence could be beneficial to their survival under natural conditions.

## 2 MATERIALS AND METHODS

### 2.1 Raw data set

A total of approximately 280 Gb of whole genome sequencing (WGS) data for Asian *O. rufipogon* and *O. sativa* genotypes were downloaded from the EMBL database; these had been generated based on paired‐end libraries (Huang et al., 2012; Wang et al., 2018). The whole genome coverage depth of sequence reads ranged from 1.02× to 7.35×, with an average of 2.53×. In all, 590 samples comprising 221 *O. rufipogon* accessions and 369 *O. sativa* cultivars, which originated from 63 countries or regions in Asia, Europe, Africa, America and Oceania, were included (Figure S1, Table S1). The samples were first selected randomly from the original data sets and then were adjusted artificially according to their geographic distribution. Among them, 91 samples, including 59 *O. rufipogon* accessions and 32 *O. sativa* varieties, were identified as containing homologous structures of *orf79* (Table S2). Five types of *O. sativa* were classified as Indica, Japonica, Aus, Aromatic and Intermediate (Wang et al., 2018).

### 2.2 The de novo assembly and annotation of draft mitochondrial genomes

The raw WGS data of 590 accessions were processed using FastQC v0.11.5 and NGSQCToolkit v2.3 software to control sequence quality of the original data set; they were then filtered using BWA and SAMtools (Li & Durbin, 2010) software to extract mitochondrial‐original reads that were properly paired to the reference mitochondrial genomes. These reads were finally used to conduct de novo assembly of the mitochondrial genomes by using SPAdes software (Bankevich et al., 2012). In order to improve the assembly quality for mitochondrial genes from the low‐coverage WGS data, we applied 4 strategies: (a) quality control reports for all paired raw data were first generated using FastQC software. The data that passed quality control on per base sequence quality, per tile sequence quality and adopter content were selected. The remaining data were further trimmed and processed in NGSQCToolkit v2.3.3 with default options except qualCutOff = 25 and cutOffQualScor = 25. (b) The interruption of excessive variation from mitochondrial genomes of distant species to this extraction was reduced by using only 13 mitochondrial genomes in the GenBank database, including 10 from *Oryza* genus, as reference genomes. The reads that paired properly and had at least one of them mapped to the reference genomes (G12 option in SAMtools) were selected as targeted reads. (c) Careful option was applied to fix the assembly errors caused by mismatches and short indels, and the cov‐cutoff option was set to auto to remove residual nuclear‐original contigs according to their abnormal kmer coverage. (d) Gap‐close and additional scaffolding were further conducted for obtaining complete *atp6‐orf79*‐like structures, if necessary.

Summary statistics were calculated to evaluate the quality of the assembled mitochondrial contigs by using QUAST (Gurevich, Saveliev, Vyahhi, & Tesler, 2013). Gene annotation of the assembled mitochondrial contigs was performed using a local BLASTN program and further artificially modified in MEGA7 (Kumar, Stecher, & Tamura, 2016), when necessary. Three *atp6‐orf79*‐like sequences related to BT‐CMS (AP017386.1), LD‐CMS (AP011077.1) and HL‐CMS (Peng et al., 2010) were obtained from GenBank or published reports and were used as query sequences to annotate the homologous structures of *atp6‐orf79*; 50 complete CDSs of protein‐coding genes in the mitochondrial genome of Nipponbare were downloaded from Ensembl Plants website and used as reference sequences for the annotation of homologous genes.

### 2.3 Haplotype and genetic diversity analyses

Genealogical relationships of the identified haplotypes were inferred using a median joining method and were further virtualized in Network v5.003 (Fluxus Technology Ltd.) and Adobe Illustrator software (Adobe Systems Incorporated). Haplotype diversity, evolutionary distances based on Tajima–Nei model and population differentiation (FST) were calculated for each group of haplotypes by using DNAsp 6 (Rozas et al., 2017) and MEGA7 (Kumar et al., 2016). The principal coordinates analysis (PCoA) was conducted using GenAIEx 6.5 (Peakall & Smouse, 2012). Population diversity (π) within and between different populations was calculated and tested using pairwise differences method in Arlequin 3.5 (Excoffier & Lischer, 2010).

### 2.4 Phylogenetic analysis

To perform the phylogenetic analysis, DNA sequences of mitochondrial genes were aligned in MAFFT software (Katoh & Standley, 2013) and were manually adjusted in MEGA7 (Kumar et al., 2016). All DNA regions were aligned separately and concatenated before analyses. Phylogenetic analyses were conducted using maximum likelihood methods in IQ‐TREE software (Nguyen, Schmidt, von Haeseler, & Minh, 2014), and the Bayes information criterion was used to determine the best‐fit model for nucleotide substitution. The ultrafast bootstrap of 2,000 generations was used to exploit the best tree and its branches. The final trees were then plotted using the online tool Interactive Tree of Life (https://itol.embl.de/). Selection pressure analysis was conducted using codeml program contained in PAML 4.9 software (Yang, 1997).

### 2.5 Geographic differentiation and biogeographical inference

The spatial auto‐relationship analysis and Mantel test were conducted using SPAGeDi 1.5a (Hardy & Vekemans, 2002) and GenAIEx 6.5 (Peakall & Smouse, 2012). Geographic central feature (GCF) and geographic median centre (GMC) of a haplotype were identified based on pairwise geographic distances of genotypes containing that haplotype. The GCF was defined as the genotype that has the shortest average distance with other genotypes, whereas the GMC was defined as a theoretical coordinate that has the shortest average distance with other genotypes. Spatial kernel density for a selected haplotype was estimated using coordinates of its holders by using package MASS (Venables & Ripley, 2002) in R (Team, 2018). The geographic region for an ancestral haplotype was inferred by constructing a linear regression model based on pairwise genetic distances and pairwise geographic distance, which was modified according to the methods described by Ramachandran et al. (2005), as follows:

y=ax+b

$$x_{i}=\frac{\sum_{j=1}^{n_{i}}d_{j}}{n_{i}}$$

where *y* is the vector of genetic distances between the target haplotype and the remaining haplotypes, which was calculated using IQ‐TREE with the best‐fit model and optimal parameters; *x* is the vector of average geographic distances between candidate origin location of the target haplotype and locations of the remaining haplotypes based on coordinates information, whereas *x_i_* is the average geographic distance between candidate location of the target haplotype and locations of an *i*th haplotype; *n_i_* is the total number of genotypes containing the *i*th haplotype; *d_j_* is the geographic distance between the candidate location of a target haplotype and the location of a *j*th genotype containing the *i*th haplotype; slope *a* indicates the mutation rate of a gene along with geographic distance, whereas intercede *b* indicates the mean distribution radius of the target haplotype. The fitness coefficients of the models with coordinates of different candidate origin locations were calculated using a customized Perl script, and a hotspot region comprising all coordinates of resulted *R* ^2^ ≥ .8 was considered as the origin area of the target haplotype. Additionally, a filtered data set of haplotypes was constructed for the genetic‐spatial regression model by taking the intersection of haplotypes in genotypic and spatial groups to eliminate the interference from foreign haplotypes (i.e. haplotypes derived from other regions or originated from nontarget haplotypes).

## 3 RESULTS

### 3.1 The de novo assembly statistics of rice mitochondrial genomes

**FIGURE 1:**
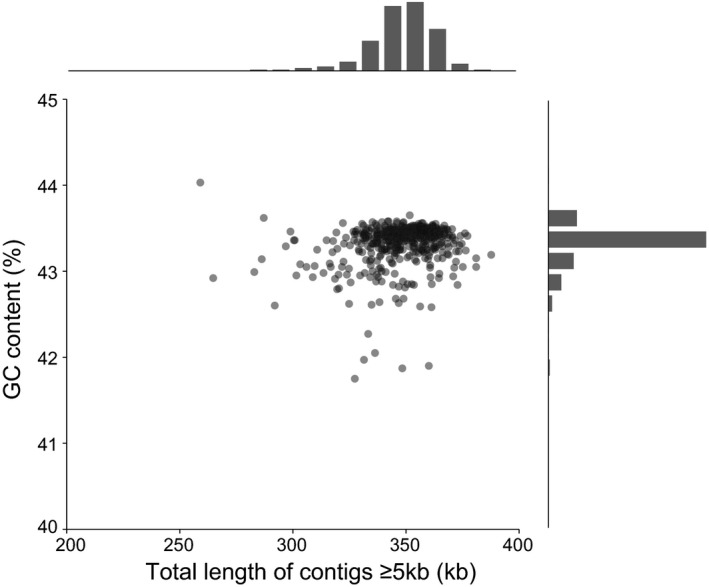
GC content and total length of long contigs in each assembled mitochondrial genome

We performed de novo assembly of the mitochondrial genomes of *O. rufipogon* and *O. sativa* based on an efficient customized pipeline. In the pre‐assembly process, a total of 314,448,811 short reads of mitochondrial origin were identified and filtered from the WGS data set of 590 genotypes as pre‐assembly data. For each genotype, an average of 532,662 mitochondrial‐original sequence reads, ranging from 124,920 to 2,555,310, were obtained using an average coverage of 111.56× for the whole mitochondrial genome, which was sufficient to assemble long genomic contigs based on the de novo approach. By using the de novo assembly pipeline, we obtained 66,581 mitochondrial contigs, with a total length of 247,771.08 kb for all the 590 genotypes; in addition, an average of 113 mitochondrial contigs with a total sequence length of 420.00 kb were obtained, with the largest contig ranging from 20.41 to 149.50 kb and having an average of 72.96 kb for each genotype. Summary statistics were calculated to evaluate the quality of the assemblies, and most of the mitochondrial‐original reads could be re‐mapped to the assembled contigs, with an average of 98.08% per genotype, ranging from 93.57% to 99.17%; the average N50 and NG50 sizes were 33.67 and 25.09 kb, ranging from 7.11 to 89.12 kb and from 5.72 to 56.48 kb, respectively (Table S3). The GC content of all assembled genomes was stable and showed an average of 43.34%, ranging from 41.75% to 44.03%. The most assembled sequences (348.64 kb/420.00 kb for an average) were located in the long contigs (≥5.00 kb) that have sufficient length to cover the most mitochondrial protein‐coding genes, including the *atp6*‐*orf79*‐like structures (Figure 1). Annotation of the mitochondrial contigs indicated that 27 protein‐coding genes, including *atp6*, were present in the form of complete coding sequences (CDS) in a single contig of all the 590 genotypes. Further, the CDSs of all the identified *orf79* alleles were also found within single contigs. These results indicated that the assembled mitochondrial genomes could be adequately used for the identification and variation analysis of *atp6‐orf79*‐like structures and other protein‐coding genes.

### 3.2 Identification of different *atp6*‐*orf79*‐like structures

To investigate sequence variation and distribution of *atp6*‐*orf79*, we screened the homologous structures in the assembled mitochondrial contigs of 590 different rice strains, including 221 *O. rufipogon* and 369 *O. sativa* accessions. A total of 91 genotypes were confirmed to contain homologous genes related to *orf79*, and 74 of them contained a homologous gene downstream of *atp6,* and formed complete *atp6*‐*orf79*‐like structures. In contrast, no *orf79* alleles or mitochondrial genomic fragments similar to *orf79* were detected in the retained 499 genotypes.

**FIGURE 2:**
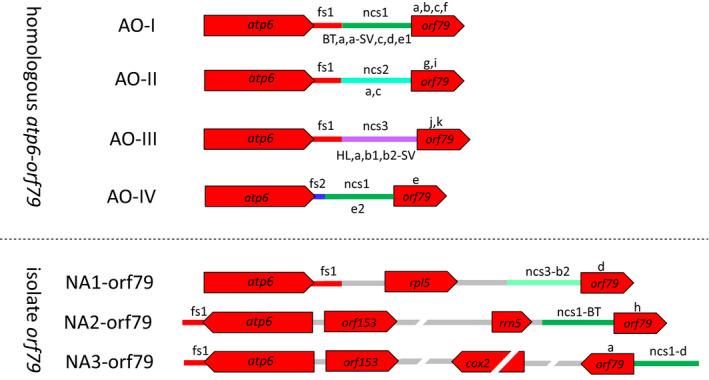
Annotation of the gametophytic CMS‐related *atp6*‐*orf79* and its homologous structures. The *atp6*‐*orf79*‐like structures consisted of four segments: *atp6* and its downstream flanking sequence (fs1 and fs2), the intergenic noncoding sequence (ncs1, ns2, ncs3) and *orf79*. Lowercase letters beside the segments denote the haplotypes of that segment. According to the variations, the *atp6*‐*orf79*‐like structures can be grouped into 4 types: group atp6‐orf79‐I (AO‐I), group atp6‐orf79‐II (AO‐II), group atp6‐orf79‐III (AO‐III) and group atp6‐orf79‐IV (AO‐IV).

A total of 16 *atp6*‐*orf79*‐like structures (H1–H16), including the previously reported BT‐CMS‐related *B‐atp6‐orf79* (H1), HL‐CMS‐related *atp6*‐*orfH79* (H11) and LD‐CMS‐related *atp6*‐*L‐orf79* (H4), were identified, whereas the remaining 13 structures were different from those reported previously (Table 1). We analysed the sequence of the 16 *atp6*‐*orf79*‐like structures by searching on GenBank (http://www.ncbi.nlm.nih.gov/) and re‐annotated their sequence compositions. The *atp6*‐*orf79*‐like structures can be divided into the following four segments (Figures 2 and S2): (1) *atp6* coding sequence, (2) the partial downstream flanking sequence (fs) of *atp6* (fs1 or fs2), (3) the intergenic noncoding sequence (ncs) containing three types of large block substitutions (ncs‐1, ncs‐2 and ncs‐3) between fs1/fs2 and *orf79*, and (4) the *orf79* coding sequence. The fs region contained a large block substitution caused by two long heterologous sequences with a length of 105 and 49 bp in fs1 and fs2, respectively, while the ncs region contained three long heterologous sequences with a length of 45, 46–55 and 51–53 bp in ncs1, ncs2 and ncs3, respectively (Table 2). The fs1 was highly conserved as the downstream region of *atp6* in most of the accessions (64/74), whereas fs2 was only detected in 10 accessions. The segments of ncs‐1 and ncs‐3 showed a high degree of homology with the upstream flanking sequence of *trnfM* and *rps7*, respectively, which are conserved in the mitochondrial genomes of *Oryza* species and other plants (such as maize, sorghum, wheat and soya bean). However, the segments of ncs‐2 exhibit little similarity to any known mitochondrial or nuclear genomic sequences. Based on the features of the 4 segments, the *atp6*‐*orf79*‐like structures can be classified into the following four groups: AO‐I (*atp6*‐fs1‐ncs1‐*orf79*, H1‐H9), AO‐II (*atp6*‐fs1‐ncs2‐*orf79*, H14‐H15), AO‐III (*atp6*‐fs1‐ncs3‐*orf79*, H10‐H13) and AO‐IV (*atp6*‐fs2‐ncs1‐*orf79*, H16). In addition, we found another three structures (NA1‐orf79, NA2‐orf79 and NA3‐orf79), among which *orf79* was located far away from *atp6* in the mitochondrial genome, and the former two structures were located in the region downstream of *rpl5* and *rrn5*, respectively (Figure 2).

**TABLE 1:** Structure composition of the 16 haplotypes detected in 74 *Oryza rufipogon* and *O. sativa* genotypes

Haplotypes	Structure compositions				Genotypes	
	Locus1−1005^a^ (*atp6*)	Locus1006−1115 (fs)	Locus1116−1221 (ncs)	Locus1222−1461 (*orf79*)	*Oryza rufipogon*	*Oryza sativa*
H1	*atp6*	fs1a	ncs1‐BT	*orf79a*	5	1
H2	*atp6*	fs1a	ncs1‐c	*orf79a*	1	20
H3	*atp6*	fs1a	ncs1‐a	*orf79a*	1	0
H4	*atp6*	fs1a	ncs1‐a	*orf79b*	1	4
H5	*atp6*	fs1a	ncs1‐a‐SV	*orf79a*	1	0
H6	*atp6*	fs1a	ncs1‐d	*orf79a*	4	4
H7	*atp6*	fs1b	ncs1‐d	*orf79a*	0	1
H8	*atp6*	fs1a	ncs1‐e1	*orf79c*	1	0
H9	*atp6*	fs1a	ncs1‐e1	*orf79f*	2	0
H10	*atp6*	fs1a	ncs3‐a	*orf79j*	2	0
H11	*atp6*	fs1a	ncs3‐HL	*orf79k*	6	0
H12	*atp6*	fs1a	ncs3‐b1	*orf79k*	3	0
H13	*atp6*	fs1a	ncs3‐b2‐SV	*orf79k*	3	0
H14	*atp6*	fs1a	ncs2‐a	*orf79g*	3	0
H15	*atp6*	fs1a	ncs2‐c	*orf79i*	1	0
H16	*atp6*	fs2	ncs1‐e2	*orf79e*	10	0

^a^ Nucleotide positions of H1 were used as the reference positions for all loci.

**TABLE 2:** Nucleotide variations in 16 haplotypes of *atp6*‐*orf79*‐like structures

Reference positions^a^	Variation types	H1	H2	H3	H4	H5	H6	H7	H8	H9	H10	H11	H12	H13	H14	H15	H16
1,008	SNP	T	T	T	T	T	T	T	T	T	T	T	T	T	T	T	A
1,011–1,115	Sub.	105	105	105	105	105	105	105	105	105	105	105	105	105	105	105	49
1,063	SNP	C	C	C	C	C	C	G	C	C	C	C	C	C	C	C	C
1,123	SNP	T	T	T	T	C	T	T	T	T	T	T	T	T	T	T	T
1,125	Ins.												T	T			
1,128–1,136	Sub.	9	9	9	9	12	9	9	9	9	9	9	9	9	9	9	9
1,149	SNP	A	A	A	A	A	A	A	A	A	A	A	A	A	G	G	A
1,153	SNP	A	A	A	A	A	A	A	A	A	G	G	G	G	A	A	A
1,159–1,203	Sub.	45	45	45	45	45	45	45	45	45	51	51	51	53	46	55	45
1,206	SNP	A	A	A	A	A	A	A	C	C	C	C	C	C	C	C	C
1,210	SNP	T	T	T	T	T	T	T	T	T	T	T	T	T	C	T	T
1,214	Ins.			4	4	4											
1,214	SNP	A	A	A	A	A	T	T	A	A	A	C	C	C	A	A	C
1,215	SNP	T	T	T	T	T	T	T	T	T	T	T	T	T	T	G	T
1,218	SNP	T	T	T	T	T	T	T	T	T	T	T	T	C	C	T	T
1,219	SNP	G	G	G	G	G	G	G	G	G	G	G	G	C	G	G	G
1,220	SNP	C	T	C	C	C	C	C	C	C	C	C	C	C	C	C	C
1,225	SNP	G	G	G	G	G	G	G	G	G	A	A	A	A	A	A	G
1,234	SNP	G	G	G	G	G	G	G	G	G	C	C	C	C	G	G	G
1,363	SNP	A	A	A	A	A	A	A	A	A	T	T	T	T	A	G	C
1,367	SNP	A	A	A	C	A	A	A	C	C	A	A	A	A	A	A	C
1,368	SNP	A	A	A	A	A	A	A	T	T	C	C	C	C	A	A	T
1,399	SNP	C	C	C	C	C	C	C	C	T	C	T	T	T	C	C	C

Abbreviations: Ins., small insertions; SNP, single nucleotide polymorphisms; Sub., block substitutions.

^a^ Nucleotide positions of H1 were used as the reference positions for all loci.

### 3.3 Haplotype analysis of the *atp6*‐*orf79*‐like structures in *O. rufipogon* and *O. sativa*

**FIGURE 3:**
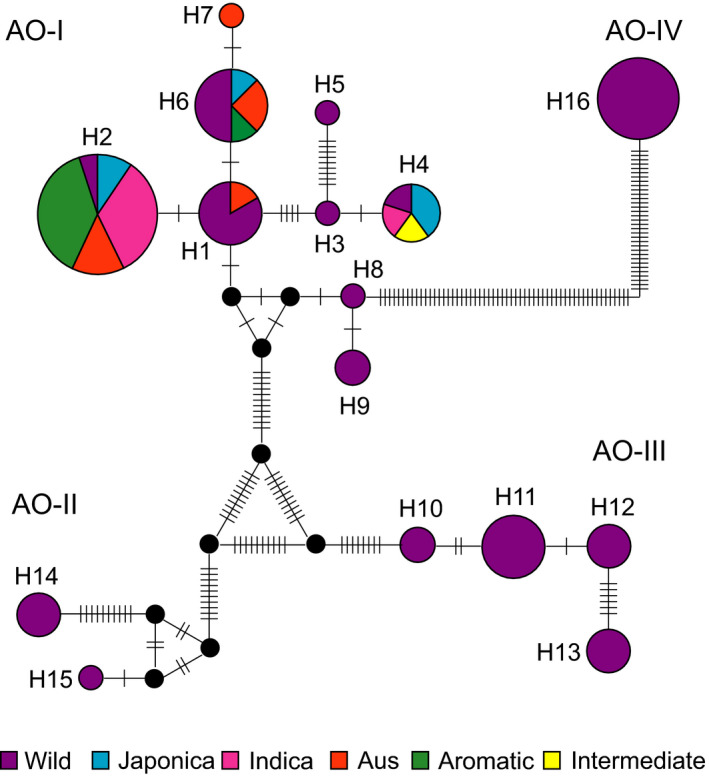
The 16 haplotypes of *atp6*‐*orf79*‐like structures detected in *Oryza rufipogon* and *O. sativa*. Each short line represents single nucleotide substitution or single nucleotide indels, and the small black dots indicate missing haplotypes (extinct or not found). The sizes of circles are approximately proportional to the sample size (*n*), with the smallest circles representing *n* = 1 and the largest representing *n* = 21

Haplotype analysis was performed on the complete sequence of the *atp6*‐*orf79*‐like structures and their compositional segments (Figures 3 and S3). Topological relationship among the different haplotypes revealed consistent results with the four groups that were classified based on their structural compositions. H1 as well as each of its structural components, including fs1a, ncs1‐BT and *orf79a*, was defined as the root node of the corresponding haplotype network for AO‐I group, suggesting that H1 is the ancestral genotype for AO‐I group (Figures 3 and S3). The most popular haplotype was H2, which was found in 21 of the 74 accessions (28.4%), followed by H16 (13.5%), H6 (10.8%), H1 (8.1%) and H11 (8.1%), whereas H3, H5, H7, H8 and H15 were identified as the rare haplotypes, each of which was detected in only one accession with a frequency of 1.4%. Among the 16 haplotypes of *atp6*‐*orf79*‐like structures—except for H7, which was only detected in a single *O. sativa* cultivar—H1, H2, H4 and H6 were found in both *O. rufipogon* and *O. sativa*, whereas the remaining 11 haplotypes only existed in *O. rufipogon* (Table 1, Figure 2).

The haplotype frequency of *atp6*‐*orf79*‐like structures was further investigated in different groups of *O. sativa* and its wild relative, *O. rufipogon* (Table S4). Most of the haplotypes (11/15) were lost in the gene flow of *atp6*‐*orf79* from *O. rufipogon* to *O. sativa*, causing a decrease in the population frequency of *atp6*‐*orf79*‐like structures from 19.9% in *O. rufipogon* to 8.1% in *O. sativa*, indicating that the *atp6*‐*orf79*‐like structures mainly differentiated along with or before the diversification of *O. rufipogon*, but only a few of them were transferred to the cultivated species, *O. sativa*, and/or an eliminating pressure was likely exerted on these pollen killer genes during domestication. For example, polymorphism of *atp6*‐*orf79‐*like structures was more abundant in the mitochondrial genomes of *O. rufipogon*, which contained 15 of the 16 haplotypes (except H7). In contrast, only 5 haplotypes in AO‐I group (H1, H2, H4, H6 and H7) were found in different *O. sativa* varieties. However, these 5 haplotypes were detected in 30 varieties of *O. sativa* with a frequency of 8.1%, whereas they were only found in 8 accessions of *O. rufipogon* with a frequency of 3.6%, indicating an increase in the gene frequency of the survivors that escaped elimination. Interestingly, their distribution in different groups of *O. sativa* also varied extensively, for instance, relatively very low frequency was found in the Japonica (2.84%) and Indica groups (6.30%), whereas exceptionally high frequency was found in the Aus (30.43%) and Aromatic groups (37.5%). These results indicated that the *atp6*‐*orf79*‐like structures were subjected to different gene flow or natural and artificial selection during the evolution and domestication of different populations of Asian cultivated rice.

### 3.4 Nucleotide variation and genetic diversity of different *atp6*‐*orf79*‐like structures

Numerous mutations were observed in the intergenic region and *orf79* coding sequence of different *atp6*‐*orf79*‐like structures. Their complete nucleotide sequences were aligned along a total length of 1,485 bp with 18 single nucleotide polymorphisms (SNPs), 3 length polymorphisms (9–105 bp) and two small insertions (1–4 bp; Table 2). Among these variations, 6 SNPs were detected in the coding region of *orf79*, whereas no variation was found in the coding region of *atp6*, indicating relatively frequent sequence variation in the CMS gene *orf79*, but high conservation of the important mitochondrial gene *atp6*. The remaining variations were all located in the intergenic region.

**FIGURE 4:**
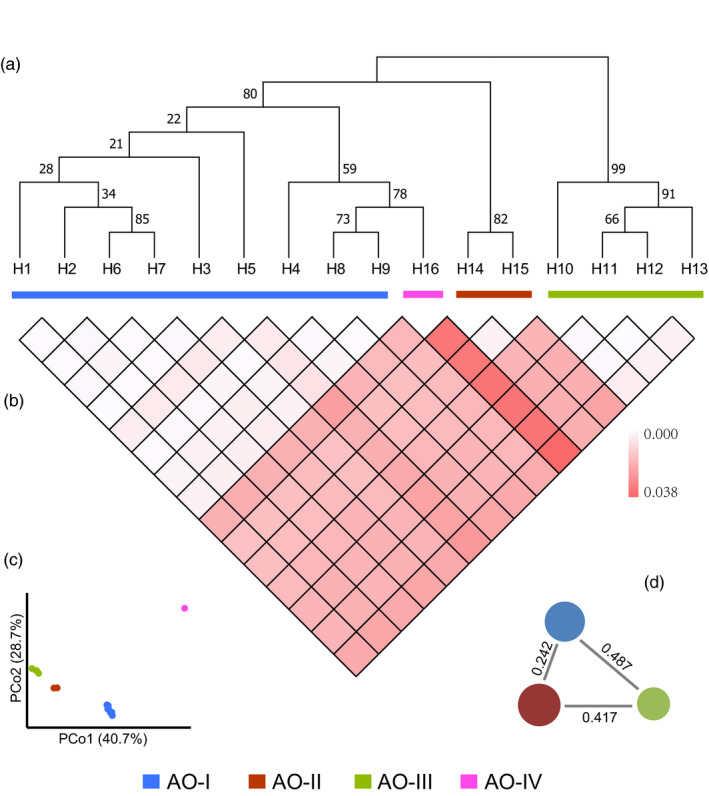
Molecular relationships among the 16 haplotypes of *atp6*‐*orf79*‐like structures. (a) Maximum likelihood tree calculated with F81 + F + I model based on nucleotide variations of *atp6*‐*orf79*‐like structures. (b) Pairwise evolutionary divergence between different haplotypes based on the Tajima–Nei model. (c) Plotting of principal coordinates analysis (PCoA) of the 16 haplotypes. (d) Illustration of genetic diversity and population differentiation in groups of OA‐I, OA‐II and OA‐III. The size of the circles represents the level of genetic diversity (*π*) of the groups, and the FST values between groups are indicated

Phylogenetic relationship and molecular diversity of haplotypes of *atp6*‐*orf79*‐like structures were assessed on the basis of the conserved regions in the complete sequences. Three main clades were identified in the phylogenetic tree—H1–H9 & H16, H10–H13 and H14–H15—which was identical to the statistical parsimony network of these haplotypes (Figures 3 and 4a). The latter two clades matched with groups AO‐II and AO‐III, respectively, and showed a classification consistent with that obtained using pairwise evolutionary distances (Figure 4b) and principal coordinates analysis (PCoA; Figure 4c). In contrast, the former clade contained two groups, AO‐I included 9 haplotypes (H1–H9), and AO‐IV included H16. AO‐I showed a relatively low genetic diversity (*π* = 0.0025) with relatively low genetic distance to AO‐II (FST = 0.242, *p* < .001), while AO‐III showed similar genetic diversity (*π* = 0.0028) but relatively higher genetic distances to AO‐I (FST = 0.487, *p* < .001) and AO‐II (*π* = 0.0017; FST = 0.417, *p* < .001; Figure 4b). These results suggested that the three distinct and independent groups of haplotypes of *atp6*‐*orf79*‐like structures—which could have been derived from different isolated ancient or intermediate genotypes—dispersed *via* independent genetic and evolutionary routes.

### 3.5 Multicentric pattern of the geographical distribution of *atp6*‐*orf79*‐like structures

**FIGURE 5:**
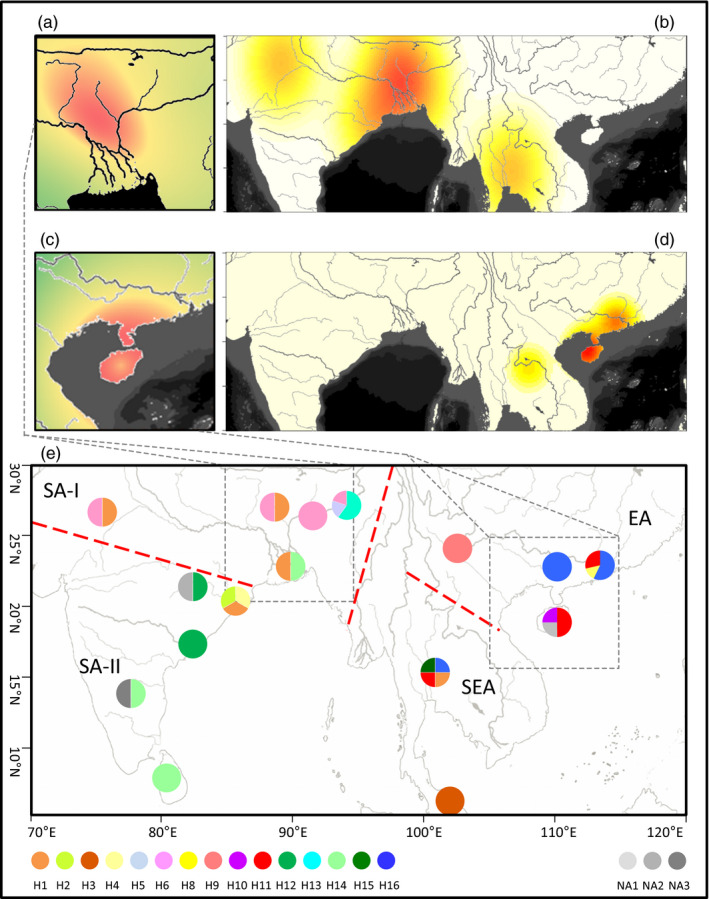
Phylogenetic and biogeographic relationships of different haplotypes of *atp6*‐*orf79*‐like structures in common wild rice. (a) The inferred origin area of H1, shown using a hotspot region of fitness in a genetic‐spatial regression model. (b) A heat map showing the distribution of probability density of H1. (c) An inferred range for the origin of H11, presented using a hotspot region of fitness in a genetic‐spatial regression model. (d) A heat map showing the distribution of probability density of H11. (e) Geographical distribution of 15 haplotypes of *atp6*‐*orf79*‐like and 3 isolate‐*orf79* structures that were detected in common wild rice accessions; the pie charts represent the proportions of different haplotypes. Colour red denotes a high value of probability density, whereas green or white denotes a low value in corresponding regions of the former 4 subfigures

Mitochondrial genes usually get distributed based on their maternally inherited gene flows with slow drift; thus, the centre of their geographical distribution could serve as an important clue to their area of origin. We investigated the geographical distribution of 15 haplotypes (except for H7 which was only detected in Asian cultivated rice varieties) of *atp6*‐*orf79*‐like structures in 44 common wild rice accessions derived from different countries or regions in East Asia (China), South‐East Asia and South Asia (Figure 5). In all, 4 regions (SA‐I and SA‐II for South Asia, EA for East Asia and SEA for South‐East Asia) were defined based on the geographical distribution of common wild rice along the major rivers (Figure 5e). Among the 15 haplotypes, H1 was the most widely distributed haplotype with an average pairwise geographical distance of 1,388.38 km, ranging from 417.86 to 1,946.53 km, having been dispersed in three regions, SA‐I (3), SA‐II (1) and SEA (1); its geographic central feature (GCF) and geographic median centre (GMC) were both located in SA‐I. It was followed by H14, with an average pairwise geographical distance of 1,416.04 km; it was detected in SA‐I (1) and SA‐II (2) regions with its GCF and GMC coordinates both in SA‐II (Table S5). H11 and H16 were detected in EA (5 and 1, respectively) and SEA (9 and 1, respectively) regions. They had an average pairwise geographical distance of 509.93 km (range, 0–1,185.82 km) and 574.39 km (range, 0–1,726.00 km), respectively, and their GCF and GMC coordinates were both located in EA (Table S5). Other haplotypes were located in a single region; for example, H2, H4 and H12 were only detected in the SA‐II region; H5 and H6 were specific to the SA‐I region; H8, H9 and H10 were found only in the EA region; and H3 was noted in the SEA region (Figure 5).

The multicentric characteristics of these haplotypes could be further summarized on the basis of their geographical distributions in the 4 regions (Figure 5; Table 3). Two distinct distribution areas were observed, SA (SA‐I and SA‐II) and EA regions contained 13 of the 15 haplotypes, but did not share any common haplotypes between them. The SA‐I region was a major distribution centre with the highest frequency of *atp6*‐*orf79*‐like structures (0.32) and *orf79* alleles (0.51) (Table 3) and covered the GCF and GMC locations of the two widely distributed haplotypes (H1 and H6) in group AO‐I (Table S5), as well as the most popular *orf79* allele (*orf79a*) in *O. rufipogon* (Table S6). SA‐II showed a relatively lower frequency of *atp6*‐*orf79*‐like structures (0.21) and *orf79* alleles (0.32) and covered the GCF and GMC locations of 4 haplotypes, including H2 and H4 in group AO‐I. The EA region was another major distribution centre with high frequencies of *atp6*‐*orf79*‐like structures (0.24) and *orf79* alleles (0.27) and encompassed the GCF and GMC locations of 5 haplotypes, including the two most popular haplotypes (H11 and H16) in AO‐III and AO‐IV, respectively, as well as 6 of the 11 *orf79* alleles. Finally, the SEA showed an exceptionally low frequency of both *atp6*‐*orf79*‐like structures (0.07) and *orf79* alleles (0.10) and shared 1 and 2 haplotypes with SA and EA, respectively, indicating that it could have served as an intermediate spreading region between the two major distribution areas.

**TABLE 3:** Detection of haplotypes of *atp6*‐*orf79*‐like structures in common wild rice accessions derived from different regions

Regions	No. of accessions	*atp6‐orf79*‐like structures				*orf79* alleles			
		No. of accessions containing a haplotype	No. of haplotypes	No. of region‐specific haplotypes	Frequency	No. of accessions containing *orf79*	No. of alleles	No. of region‐specific alleles	Frequency
SA‐I	37	12	5	3	0.32	19	3	0	0.51
SA‐II	38	8	5	3	0.21	12	5	1	0.32
EA	79	19	5	3	0.24	21	6	4	0.27
SEA	67	5	5	2	0.07	7	4	1	0.10
Average	49.25	11	5	2.75	0.23	14.75	4.5	1.5	0.32

Nevertheless, geographical isolation or barrier could have also affected the distribution of *atp6*‐*orf79*‐like structures; for example, a total of 11 haplotypes were region‐specific, of which 3, 3, 3 and 2 haplotypes were region‐specific in the SA‐I, SA‐II, EA and SEA regions, respectively.

### 3.6 Spatial‐genetic regression confirms the pattern of origin of the *atp6*‐*orf79*‐like structures

The disturbances associated with multicentric distribution and geographical factors resulted in a nonsignificant correlation (*R* = . 48, *p* = .184) between genetic and geographical distances with respect to the haplotypes of *atp6*‐*orf79*‐like structures from all four regions in the Mantel test, which is not difficult to understand: two accessions possessing the same haplotype can be located far away owing to geographical dispersal of the same haplotype, and accessions with different haplotypes can also coexist in the same region owing to the founder effect of haplotypes from different areas of origin, indicating that the *atp6*‐*orf79*‐like structures had a complex evolutionary pattern.

Therefore, we constructed a spatial‐genetic regression model to infer the centre of origin of a specific haplotype. The area of origin of H1 was ascertained on the basis of an intersectional data set of haplotypes in genotypic (AO‐I, AO‐II) and spatial groups (SA regions), which consisted of 6 haplotypes of *atp6*‐*orf79*‐like structures—H1, H2, H4, H5, H6 and H14. The Mantel test revealed a significant correlation (*R* = .442, *p* = .027) between the genetic and geographical distances of these haplotypes and indicated a significant spatial structure. Therefore, a linear genetic‐spatial regression model for H1 was further constructed on the basis of pairwise genetic and geographical distances, and an apparent hotspot region was identified with high fitness coefficients (*R* ^2^ ≥ .8). This hotspot region (23.7°N–27.6°N and 86.7°E–90.4°E) was located in north‐east of Gangetic Plain on the Indian Peninsula, which was surrounded by five rivers, that is Koshi River, Ganges River, Padma River, Brahmaputra River and Teesta River, in the SA‐I region (Figure 5a) and covered the GCF (23.7°N, 90.4°E) and GMC coordinates (23.7°N, 89.1°E) of H1. This region was further confirmed by a consistent hotspot region with high probability density of geographical distribution for H1 (Figure 5b). These evidences strongly suggested that the centre of origin of the BT‐CMS‐related H1 was located in this hotspot region. Thus, H1 could be concluded to have originated from this region and later spread out, evolving into other 5 or more haplotypes, including the LD‐CMS‐related H4.

A rough geographical range was obtained for the area of origin of HL‐CMS‐related H11 based on a small data set comprising intersectional haplotypes H11 and H10 between genotypic (AO‐III) and spatial groups (EA and SEA regions) and an external H1. The results from the genetic‐spatial regression model revealed a relatively wide hotspot area in South China, including Hainan Island and Leizhou Peninsula (Figure 5c). This region of origin was further supported by the GCF coordinate (19.5°N, 109.5°E) and the resulting hotspot region of probability density of geographical distribution for H11, both of which were located in Hainan Island of China (Figure 5d).

### 3.7 Gene flow of *orf79* alleles in the mitochondrial genomes of *O. rufipogon* and *O. sativa* species

**FIGURE 6:**
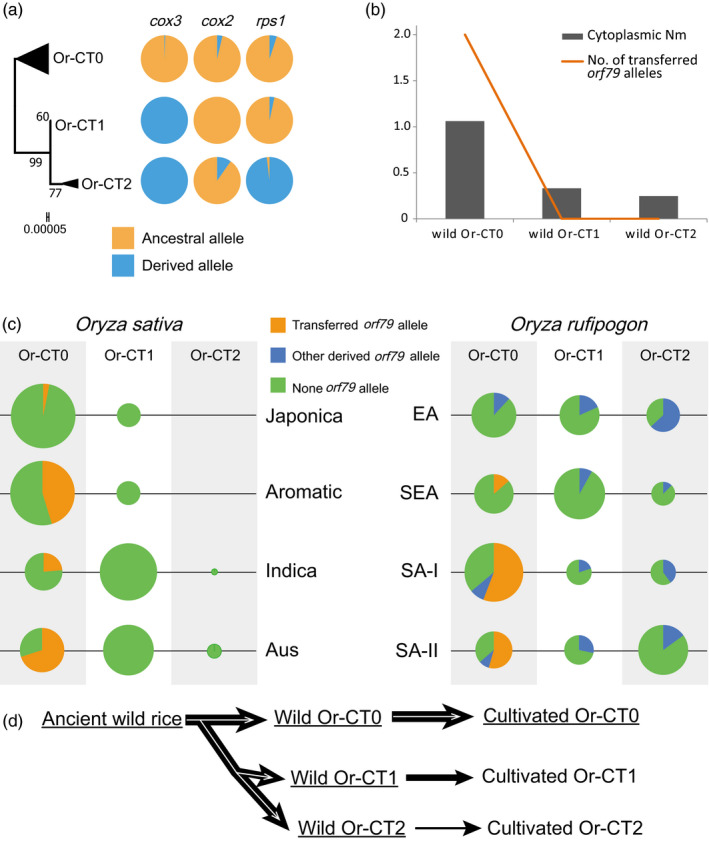
Gene flow of *orf79* alleles in the mitochondrial genomes of common wild rice and Asian cultivated rice. (a) The spectrum of allele frequencies at the causal polymorphisms of *cox3*, *cox2* and *rps1*. Their ancestral alleles were identified using an outgroup species *Oryza meridionalis*. (b) Mitochondrial gene flow (Nm) and number of transferred *orf79* alleles between cultivated rice and wild rice populations with different cytoplasm. (c) Frequency changes of *orf79* alleles detected in *O. sativa* and *O. rufipogon* accessions. The areas of circles were determined using sample proportion (*p*) in each population, with the smallest circles representing *p* = 0.79% and the largest representing *p* = 88.07%. Two ancestral alleles *orf79a* (also detected in an outgroup species *Oryza barthii*) and *orf79b* were transferred into *O. sativa* from *O. rufipogon*, whereas the remaining alleles were absent in *O. sativa*. (d) Schematics of the gene flow of *orf79* alleles along with the flowing of mitochondrial genomes in *O. rufipogon* and *O. sativa* species. The black arrows denote the flowing of mitochondrial genomes in the entire rice populations, whereas the white arrows denote the gene flow of *orf79* alleles in some subpopulations (highlighted by underline)

Since the *orf79* alleles flowed only in some subpopulations as a part of mitochondrial gene flows of the entire populations, we investigated the cytoplasmic phylogenetic relationship of all 590 *O. rufipogon* and *O. sativa* accessions. Three cytoplasmic types, that is Or‐CT0, Or‐CT1 and Or‐CT2, were defined according to the maximum likelihood tree that was generated based on nucleotide variations of 3 variable mitochondrial genes (*cox3*, *cox2* and *rps1*) in all tested accessions and an outgroup species *Oryza meridionalis* (Figure 6a, Table S7). Among them, Or‐CT0 contained most of the ancestral alleles of *cox3* (99.40%), *cox2* (96.39%) and *rps1* (94.86%), followed by Or‐CT1 and Or‐CT2, indicating three distinct routes of evolution of the mitochondrial genomes in *O. rufipogon* and *O. sativa*.

Mitochondrial gene flows differed between *O. sativa* and the three cytoplasmic types of *O. rufipogon*, as revealed by the data sets of all accessions and the subset of accessions containing *orf79* alleles (Figure 6b,c). A considerably more frequent mitochondrial gene flow was detected between cultivated rice and the Or‐CT0 type of wild rice (Nm = 1.062, Fst = 0.320) than that between cultivated rice and the Or‐CT1 and Or‐CT2 types of wild rice populations (Nm = 0.333, Fst = 0.600 and Nm = 0.248, Fst = 0.669, respectively), as represented by the 3 variable mitochondrial genes. Accordingly, the vertical transfer of two *orf79* alleles, *orf79a* and *orf79b*, from the mitochondrial genomes of wild Or‐CT0 (with a frequency of 31.91% for *orf79* alleles) to cultivated Or‐CT0 (13.50%) was detected, but no transfer of *orf79* alleles was observed from Or‐CT1 and Or‐CT2 types of wild rice (14.67% and 34.62%, respectively) to the relative types of cultivated rice (Figure 6b–d).

Furthermore, the intensities of mitochondrial gene flows from *O. rufipogon* to different populations of *O. sativa* were found to differ. Most mitochondrial genomes in Japonica and Aromatic groups (88.07% and 88.00%, respectively) had descended from the Or‐CT0 type of wild rice populations, whereas 69.29% and 54.17% of mitochondrial genomes in Indica and Aus groups had descended from Or‐CT1 type of wild rice populations, respectively (Figure 6c). Accordingly, Japonica and Aromatic groups showed considerably higher Nm (5.700 and 7.173, respectively) with Or‐CT0 type of wild rice than with the other types, whereas the latter two groups showed more frequent gene flow with wild Or‐CT1 (Nm = 1.284 and 0.820, respectively), which further supported the conclusion that cultivated rice, especially Japonica and Aromatic groups, showed a considerably frequent mitochondrial gene flow with Or‐CT0 type of wild rice. However, the frequencies of *orf79* alleles in the Or‐CT0 type of domesticated groups did not correspond with the intensity of wild‐to‐cultivated gene flows in this type; for example, an exceptional low frequency of the transferred *orf79* alleles was observed in the Or‐CT0 type of Japonica group (3.23%), whereas it was relatively high in the Or‐CT0 type of the other three domesticated groups—Indica (23.68%), Aromatic (45.45%) and Aus (70.00%)—indicating the complex and special gene flows of these mitochondrial CMS genes. Moreover, wild Or‐CT0 populations in SA‐I and SA‐II regions showed considerably higher frequencies of *orf79* alleles (54.55% and 51.85%, respectively) than that in SEA (13.64%) and EA (0%), and almost all transferred *orf79* alleles and *atp6‐orf79*‐like structures were detected in the SA regions, although a few of them were identified in SEA (Figure S5), strongly suggesting that the transferred *orf79* alleles in the 4 groups of cultivated rice had mostly descended from the wild rice populations in the SA region.

## 4 DISCUSSION

### 4.1 An efficient pipeline for the auto‐assembly of mitochondrial genes based on low‐coverage WGS data

As an organelle genome of endosymbiotic acquisition and maternal inheritance, the mitochondrial genome plays a very important role in studies related to productivity and development (Ogihara et al., 2005), evolution and historical phylogeny of plants (Lonsdale, 1988; Palmer & Herbon, 1988; Wolfe et al., 1987; Ye et al., 2017), as well as those related to variation, exchange and interaction between functional genes in nuclear and mitochondrial genomes (Hsu & Mullin, 1989; Knoop, 2004; Stern & Lonsdale, 1982; Warren, Simmons, Wu, & Sloan, 2016). The number of completed mitochondrial genomes has been increasing over the past few years, and hundreds of plant mitochondrial genomes have been deposited in the GenBank database. However, assessing completed mitochondrial genomes based on sequencing data created using second‐ and/or third‐generation sequencing platforms is both time‐ and resource‐consuming (Notsu et al., 2002; Shi et al., 2018), which makes it difficult to analyse mitochondrial genes on a large scale. Alternatively, Sanger sequencing of cloned fragments amplified using PCR or special PCR methods, for example hiTAIL‐PCR (Jaramillo‐Correa, Aguirre‐Planter, Eguiarte, Khasa, & Bousquet, 2013; Luan et al., 2013; Tang et al., 2017), is commonly used, although it is also a laborious process.

In this study, we constructed an efficient pipeline to facilitate batching de novo assembly of long mitochondrial scaffolds for assessing mitochondrial genes from low‐coverage WGS data of large collection. The main purpose of our pipeline was to assemble each of the CDS regions of mitochondrial genes in a single contig irrespective of the length; with this approach, we could produce accurate and continuous sequences of mitochondrial genes by avoiding interruption with abundant structural variations of noncoding regions and contamination of nuclear genomic fragments. Several strategies were employed to improve the continuity while ensuring the accuracy of the assembled mitochondrial genomes, for example by using an optimal number of 13 mitochondrial genomes (10 from *Oryza* genus and 3 from other relative species of Poaceae) instead of going by the common practice of using a collection of dozens of different species or even all plant species as reference genomes; this enhanced the efficiency of filtering of the target mitochondrial‐original reads from the WGS data set and reduced nontarget mapping resulting from incorporation of excessive sequence variations from reference genomes of unrelated species. When the assembly was performed using SPAdes software, different kmer values were automatically tested to obtain an optimal kmer, and the Mismatch Corrector module was activated to fix the errors in assembly caused by mismatches and short indels; the residual nucleus‐ and plastid‐originated contigs were further identified and removed based on their abnormal coverage depth (e.g. coverage depth of mitochondrial contigs was usually tens or even more than one‐hundred‐fold that of nuclear contigs, but less than one‐third that of plastid contigs). By using this pipeline, we could assemble draft mitochondrial genomes of 590 genotypes with an average coverage depth of 112.85×, which was considerably higher than that of the nuclear genome (2.53×) in the original WGS data set. In addition to the CMS gene *orf79*, 27 distinct mitochondrial genes, including 20 conserved genes, were successfully assembled in a single contig from low‐coverage WGS data set (Table S7). The nucleotide sequences of these 20 genes were highly conserved and remained unchanged in all 590 assembled genomes, which further underscored the reliability of this pipeline (Table S7). However, additional manual scaffolding was needed to generate continuous CDSs of some nonconserved mitochondrial genes and intergenic sequences owing to abundant variations in those regions; for example, 14 mitochondrial protein‐coding genes—in the more than 200 genotypes—were annotated with their CDS regions split into more than one contig, which could still be fixed manually, but would be a time‐consuming process.

### 4.2 Multi‐origination and complex diversification process of *atp6*‐*orf79*‐like structures during the co‐evolution of common wild rice and Asian cultivated rice

We found that (a) both the ancestral haplotype (H1) of *atp6‐orf79*‐like structures and the ancestral allele (*orf79a*) of *orf79* in group AO‐I were the most detected in *O. rufipogon* accessions derived from SA‐I, with a frequency of 11.11% and 51.85%, respectively, followed by that in SA‐II (9.09% and 45.45%, respectively), SEA (4.55% and 13.64%, respectively) and EA (0% and 0%, respectively), indicating their clear flow from SA regions to SEA and finally likely to EA; (b) ancestral haplotypes in AO‐II (i.e. H10 and H11) and AO‐IV (i.e. H16) groups showed a dispersing tendency from EA (with higher frequency) to SEA and then to SA regions (with lower frequency); (c) many haplotypes showed cytoplasmic‐ or region‐specific distribution; for example, H14 was distributed only in Or‐CT1 type of *O. rufipogon* accessions, whereas *orf79e* was limited to Or‐CT2 type of *O. rufipogon* accessions, and 11 of the 15 *atp6*‐*orf79*‐like structures as well as 6 out of 11 *orf79* alleles were region‐specific; (d) a total of 8 *atp6*‐*orf79*‐like structures and 6 *orf79* alleles were detected in the ancestral Or‐CT0 type of *O. rufipogon*, suggesting that the divergence of these CMS‐related structures may have occurred before the origin of *O. rufipogon* (Fig. S4). These results indicated a multi‐original development of the *atp6*‐*orf79*‐like structures as well as that of *orf79* alleles in *O. rufipogon*, indicating that they may have originated and diversified during or before the diversification of different populations of *O. rufipogon* in SA and EA regions, respectively, and after which they further dispersed to SEA with a flowing‐and‐varying feature. We further investigated the previously reported variation of *orf79* (Duan et al., 2007, 2015; Li et al., 2008; Luan et al., 2013) and obtained a total of 15 alleles that were all different from those identified in this study (except *orf79a* and *orf79k*). Most of them (10/15) were only detected in a single genotype, indicating a potential cytoplasmic‐ or region‐specific distribution; three alleles, including *orf79k*, were detected in the outgroup species (excluding *O. rufipogon*, *O. nivara* and *O. sativa*), for example *O. meridionalis*, *Oryza glumaepatula* and *Oryza barthii*. These results suggested a considerably early origin and diversification of *orf79* before the speciation of *O. rufipogon* and further supported our proposed multi‐origin and diversification features of the *atp6‐orf79*‐like structures.

Moreover, the gene flow model for *orf79* alleles proposed in this study provided more evidences regarding the origin and evolution of the Asian cultivated rice (Figure 6e). The evolutionary origins and domestication processes of Asian cultivated rice have long been debated in a wide range of genetic and archaeological studies, which could be broadly classified as advocating either a single or multiple origins for cultivated rice. Recent studies had concluded that Or‐IIIa group of *O. rufipogon* in South China (EA) was the ancestor of *Oryza sativa* ssp. *japonica* (Huang et al., 2012), whereas some *Oryza sativa* ssp. *indica* have been domesticated independently (Wang et al., 2018). In this study, we investigated the cytoplasmic polymorphism of 221 *O. rufipogon* accessions and 369 *O. sativa* varieties based on mitochondrial genes, including gametophytic CMS‐related *atp6‐orf79*‐like structures. We also revealed that all groups of *O. sativa* had accepted *orf79* alleles from the most ancestral Or‐CT0 type of *O. rufipogon*, but no *orf79* allele was inherited from the wild Or‐CT1 type of cytoplasm, although more than half varieties in Indica and Aus groups were identified as Or‐CT1 type. In addition, the Aromatic and Aus groups showed considerably high frequency of *atp6‐orf79*‐like structures and *orf79* alleles, which was closer or higher than that of the wild rice in SA regions. These results revealed a considerably frequent gene exchange among Asian cultivated rice and common wild rice after the early domestication of Japonica, which led to considerably higher diversification of the Asian cultivated rice and common wild rice in SA regions.

### 4.3 Strong positive selection pressure on the gametophytic CMS genes in the rice mitochondrial genome

The mitochondrial CMS genes always showed special genetic variation and evolution features, owing to their maternal inheritance and co‐existence with restorer genes, under natural conditions. Although harmful, they could be inherited with a closed CMS/Rf system that comprised a mitochondrial CMS gene conferring male sterility and one or more nuclear restorer gene(s) cancelling the male sterility. As the CMS gene was inherited only by the cytoplasmic genome, its frequency in a population mainly depended on genotypic frequencies of the corresponding restorer gene(s) and nonrestorer alleles. For example, under ideal conditions, when all individuals (*r_Rf_* = 100%) in a population contained the homozygous dominant restorer gene (*RfRf*), the CMS gene would be totally inhibited in the entire population, and its frequency (*r_cms_*) would be stable according to the Hardy − Weinberg equilibrium. In contrast, when all individuals (*r_rf_* = 100% − *r_cms_*) in a population contained the homozygous recessive nonrestorer allele (*rfrf*), different frequencies would be observed for sporophytic and gametophytic CMS genes: the frequency of sporophytic CMS genes (CMS/*RfRf*, CMS/*Rfrf* and CMS/*rfrf*) would decrease extensively owing to the sterility of progenies (CMS/*rfrf*) containing both CMS gene and *rfrf* allele, and incomplete restoration of sterility would be observed in some heterozygous progenies (CMS/*Rfrf*), whereas the frequency of gametophytic CMS genes (CMS/*RfRf*, CMS/*Rfrf*) may only decrease slightly because of the incomplete restoration of heterozygosity (CMS/*Rfrf*) of some restorer genes. When there was a dispersal of restorer genes (100% < *r_rf_* < 100% − *r_cms_*), and when they were transferred to individuals without the CMS gene, similar changes in the frequencies of CMS gene would be observed as in the previous condition, but to a lesser extent. In other words, the natural selection pressure on gametophytic CMS genes was generally much weaker than that on sporophytic CMS genes. Previous studies had reported the exertion of purifying (natural) selection pressure on sporophytic WA‐CMS genes such as *WA352* and *WA314*, both of which showed very low frequency of their alleles (*WA352a/b/c* and *WA314a/b*) in *O. rufipogon* (0.05 and 0.04, respectively) (Tang et al., 2017). In the present study, we evaluated the geographical distribution and diversification of homologous structures of the gametophytic CMS‐related *atp6*‐*orf79* and found a total of 16 *atp6*‐*orf79*‐like structures and 11 *orf79* alleles with relatively higher frequencies (0.23 and 0.32, respectively) than those of WA‐CMS genes in *O. rufipogo*n, which was consistent with the above inference.

Furthermore, we investigated the selection pressure on nucleotide variations of these *orf79* alleles during the evolutionary process (Table S8). A total of 15 nucleotide variations were identified in the *orf79* alleles that caused 12 amino acid variations with a high substitution/transversion (ts/tv) ratio of 2.201 and a large nonsynonymous‐substitution/synonymous‐substitution (dN/dS) ratio of 4.392, indicating a strong positive selection pressure on *orf79* during evolution (Tables 2 and S8). Combined analysis yielded 4 positively selected codon sites, that is scs1, scs3, scs4 and scs5, which were significant (Pr > 0.95) in both naive empirical Bayes (NEB) and Bayes empirical Bayes (BEB) analysis. The strongest selection pressure was estimated for scs3 with the dN/dS ratio of 64.928 and 9.220, both of which were significant (Pr = 1), based on NEB and BEB methods, respectively (Table S8). These results revealed that variations in these positively selective loci were encouraged and beneficial to the survival of the CMS genes. This was likely because more restorer genes were induced along with the occurrence of new alleles of the gametophytic CMS gene. *Rf1* was first reported as the restorer gene in BT‐CMS (*orf79a*) line and was shown to have the ability to restore fertility in the LD‐CMS (*orf79b*) line (Itabashi et al., 2009; Wang et al., 2006); *Rf5* and *Rf6* were found to be the restorer genes of the HL‐CMS (*orf79k*) line (Hu et al., 2012; Huang et al., 2015); *Rf2* can completely restore the fertility of the LD‐CMS (*orf79b*) line and weakly restore the fertility of the BT‐CMS line (Itabashi et al., 2009; Kazama et al., 2016). Therefore, divergence of the *orf79* alleles could broaden the resources of sterility restorer genes and facilitate the survival of the CMS genes.

### 4.4 Potential exploitation of the cytoplasm with new haplotypes of *atp6*‐*orf79*

CMS has been widely used for commercial hybrid seed production in many cereal crops such as maize and rice for decades. Hybrid rice has exhibited increased yield (by about 20%) compared with that obtained using inbred rice varieties (Cheng, Zhuang, Fan, Du, & Cao, 2007; Ma & Yuan, 2015), and has made a great contribution to the global food production (Zhu, 2016). However, potential risks exist when hybrids based on a single CMS cytoplasm are continuously grown in large areas. For instance, in 1970, an outbreak of Southern corn leaf blight (*Helminthosporium maydis* Nisikado & Miyake race T) occurred in U.S. maize hybrids produced using Texas‐type CMS that carried a mitochondrial gene T‐urfl3 with the dual role of causing CMS and disease susceptibility (Levings, 1990). Thus, the exploitation of novel CMS cytoplasms to enrich the cytoplasmic diversity is very important for commercial hybrid seed production. In this study, we screened the mitochondrial scaffolds of 590 common wild rice accessions and Asian cultivated rice cultivars and yielded 16 haplotypes of *atp6*‐*orf79*, of which H1, H4 and H11 had been previously confirmed to cause BT‐CMS, LD‐CMS and HL‐CMS, respectively (Table S2), whereas the remaining 13 haplotypes obtained here were different with previous reports. Among them, 5 haplotypes—H2, H3, H5, H6 and H7—could be considered as candidate genes conferring gametophytic BT‐like CMS because they shared identical *atp6* and *orf79* of H1, with variations only in the intergenic sequences (Table S2). Similarly, H12 and H13 could be the candidate genes conferring gametophytic HL‐like CMS. These results can provide new cytoplasmic resources for research and application of BT‐CMS and HL‐CMS. The remaining 6 haplotypes contained different *orf79* alleles with known rice CMS‐related genes and could serve as candidate genes for developing new type of CMS in rice.

In addition, although the CMS/Rf system has been widely used in global hybrid rice production, genetic resources with restorer genes for the gametophytic CMS (such as BT‐CMS, HL‐CMS and Dian1‐CMS) are still limited in rice, especially in japonica rice (Huang, Zhi‐Guo, Zhang, & Shu, 2014; Huang, 2012). In this study, a total of 74 fertile accessions, including 44 *O. rufipogon* accessions and 30 *O. sativa* varieties, were identified to have 16 haplotypes of the CMS gene *atp6*‐*orf79* and were speculated to contain the corresponding restorer genes. Seventeen genotypes, including 12 common wild rice accessions, 2 indica, 2 japonica and 1 Aus rice varieties, were identified to have the known gametophytic CMS genes (H1, H4 and H11) and corresponding restorer genes. Thirty‐eight genotypes, including 13 common wild rice accessions, 7 indica, 3 japonica, 9 Aromatic and 6 Aus rice varieties, were identified to have 5 BT‐CMS‐like and 2 Hl‐CMS‐like haplotypes of *atp6*‐*orf79*. The remaining 19 genotypes were common wild rice accessions with novel haplotypes. New restorer genes identified from these genotypes could be exploited for the gametophytic CMS/Rf system in rice.

## CONFLICT OF INTEREST

None declared.

## Supporting information

Fig S1‐S5

Table S1

Table S2‐S8

## Data Availability

Assemblies of mitochondrial genomes of the 91 accessions that contained *orf79* alleles are available in the European Nucleotide Archive (https://www.ebi.ac.uk/ena) under the study accession number PRJEB29274. And the sequences of 16 *atp6‐orf79‐like* structures (H1‐H16) and 3 structures containing isolate *orf79* allele (NA1‐orf79, NA2‐orf79 and NA3‐orf79) have been deposited to European Nucleotide Archive under the accession numbers LR794108‐LR794123 and LR794153‐LR794155.
