# Supplementary material for: Multicentric origin and diversification of atp6‐orf79‐like structures reveal mitochondrial gene flows in Oryza rufipogon and Oryza sativa
Source: Evol Appl. 2020 Jun 27;13(9):2284–99. doi: 10.1111/eva.13022 (PMC7513716; doi:10.1111/eva.13022)
Supplement: Supplementary file 1 — Fig S1‐S5 [file EVA-13-2284-s001.docx]

**Supplement figure captions**

**Fig. S1** Geographic distribution of 590 *Oryza sativa* and *Oryza rufipogon* accessions used in this study. The sizes of green circles are approximately proportional to the number of samples in that location.

**Fig. S2** The alignments of the nucleotide sequences of partial 3’ flanking sequence (fs) of *atp6*, intergenic non-coding sequence (ncs) region and *orf79* coding sequences.

**Fig. S3** Haplotype analysis on partial 3’ flanking sequence (fs) of *atp6*, intergenic non-coding sequence (ncs) region and *orf79* coding sequences, respectively.

**Fig. S4** The frequency changes of haplotypes of *atp6-orf79*-like structures and *orf79* alleles in different evolution periods and geographic regions. In each subplot, the abscissa axis referred to the three cytoplasmic types: Or-CT0, Or-CT1 and Or-CT2 while the vertical axis referred to frequency of the corresponding haplotype with a minimum value of 0 and a maximum value of 0.6.

**Fig. S5** Geographic distribution of different cytoplasmic types, *orf79* alleles and *atp6-orf79*-like structures in wild Or-CT0 populations. (a) Geographic distribution of wild Or-CT0, Or-CT1 and Or-CT2 types of accessions in South Asia, South-east Asia and East Asia; (b) Kernel density estimation of wild Or-CT0 type of accessions; (c) Geographic distribution of wild Or-CT0 type of accessions with transferred and other non-transferred *orf79* alleles and accessions without any *orf79* allele; (d) Geographic distribution of wild Or-CT0 type of accessions with transferred and other non-transferred *atp6-orf79*-like structures and accessions without any *atp6-orf79*-like structure.

**Supplement figures**

**
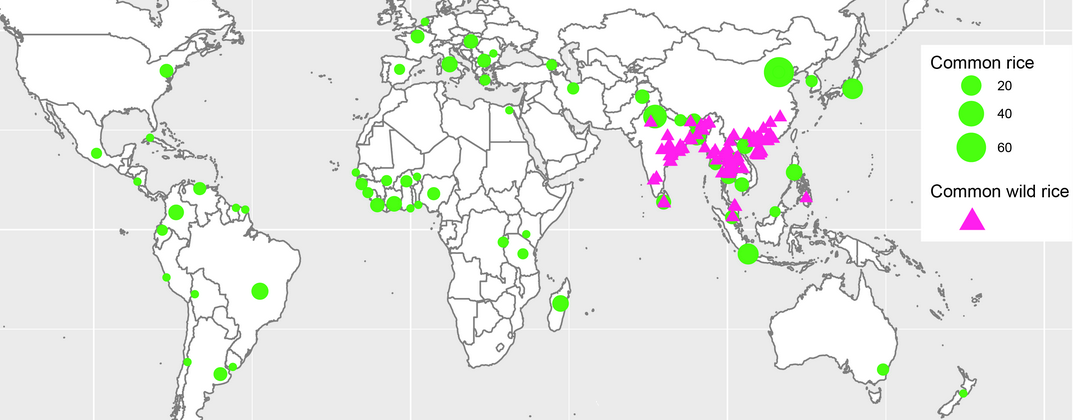
**

**Fig. S1** Geographic distribution of 590 *Oryza sativa* and *Oryza rufipogon* accessions used in this study.


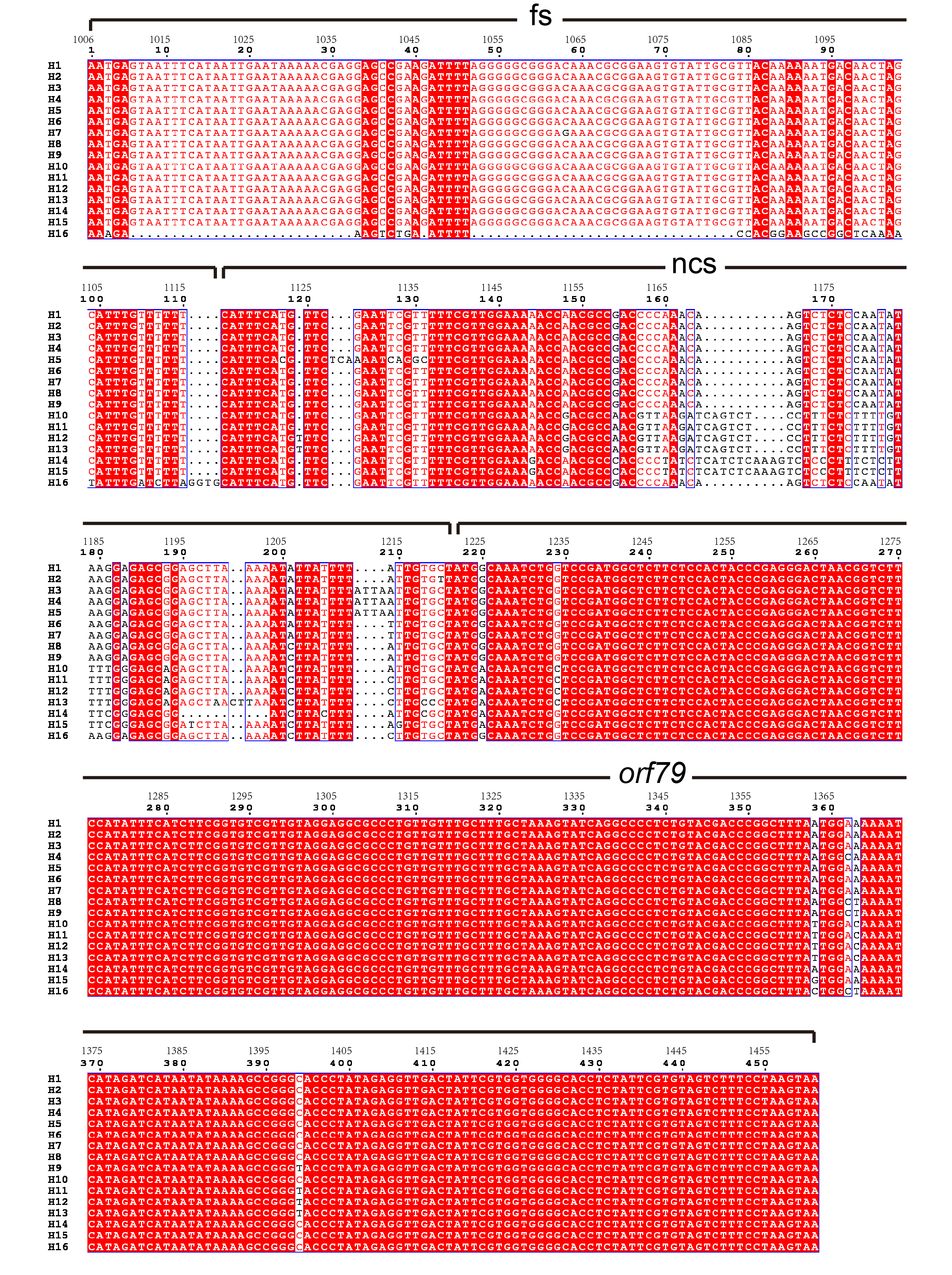


**Fig. S2** The alignments of the nucleotide sequences of partial 3’ flanking sequence (fs) of *atp6*, intergenic non-coding sequence (ncs) region and *orf79* coding sequences.


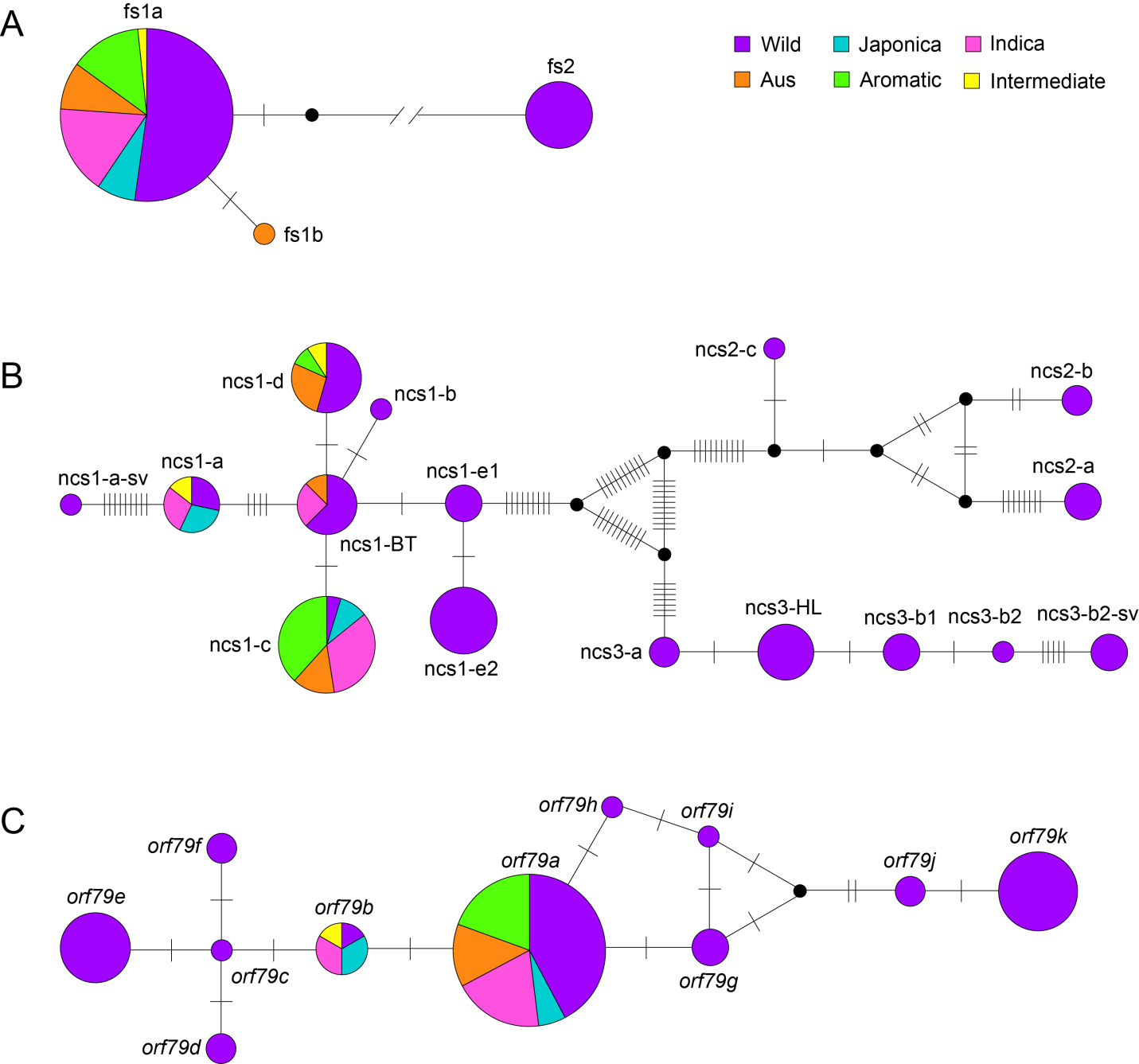


**Fig. S3** Haplotype analysis on partial 3’ flanking sequence (fs) of *atp6*, intergenic non-coding sequence (ncs) region and *orf79* coding sequences, respectively.


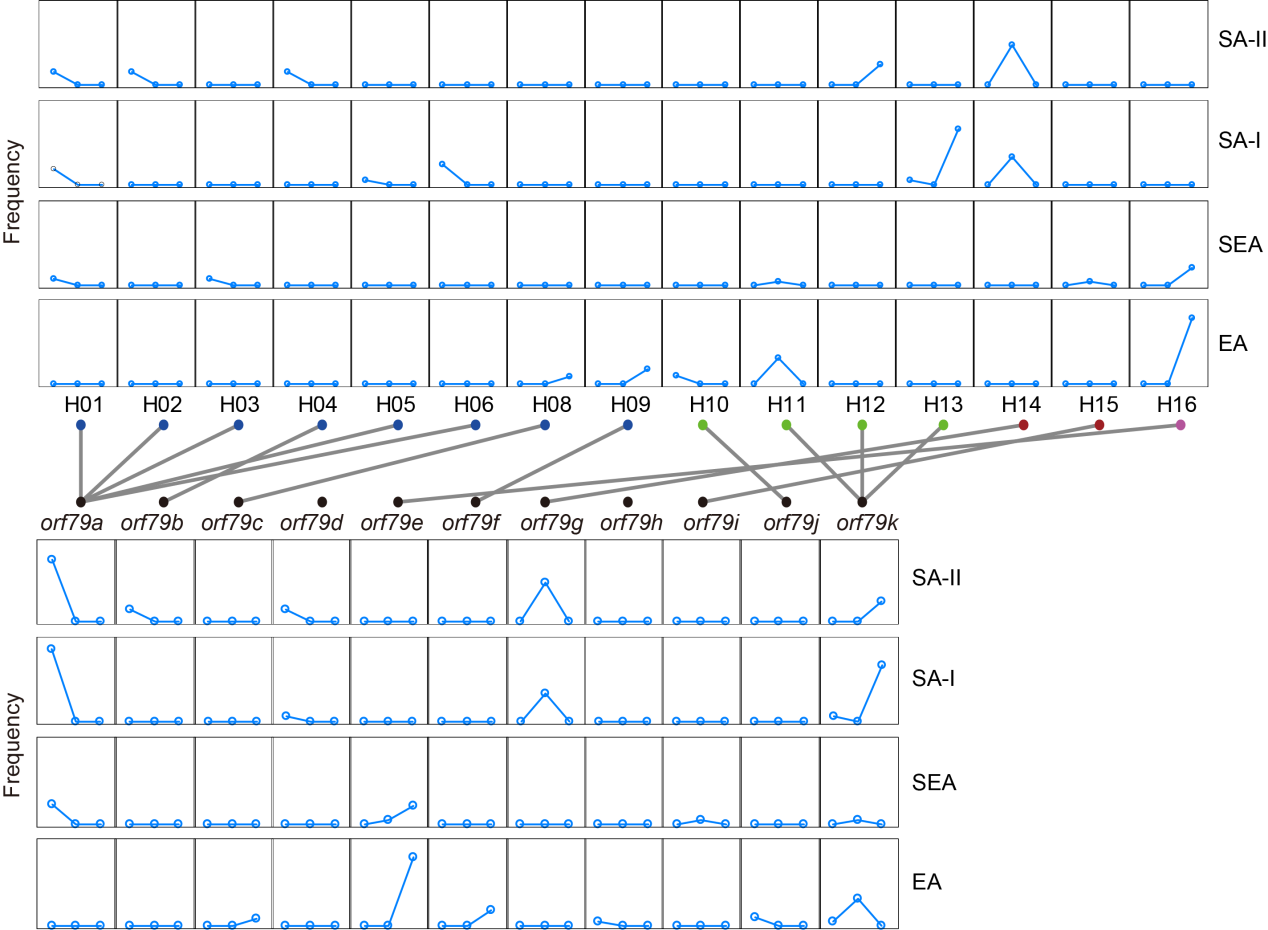


**Fig. S4** The frequency changes of haplotypes of *atp6-orf79*-like structures and *orf79* alleles in different evolution periods and geographic regions.

**
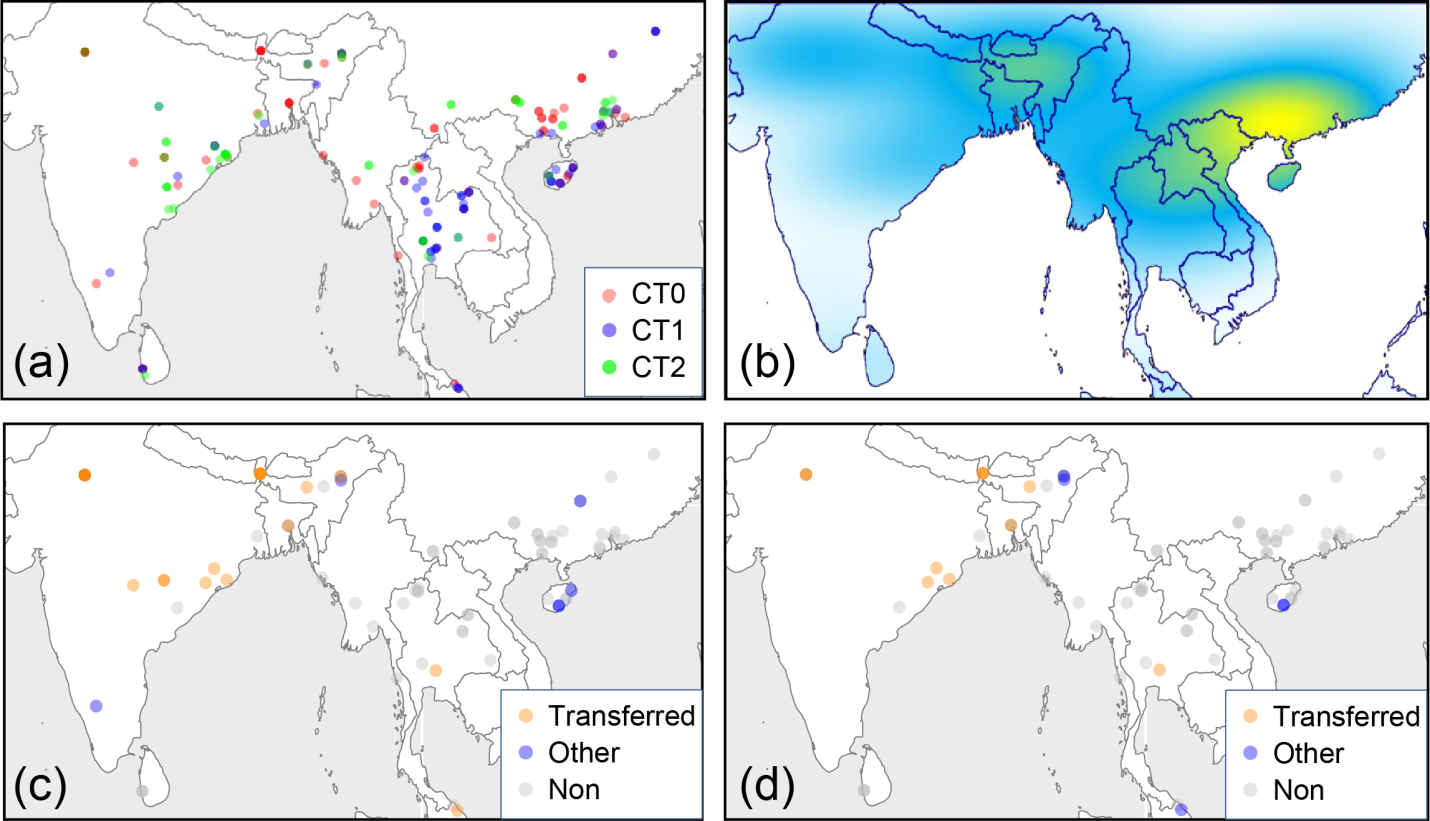
**

**Fig. S5** Geographic distribution of different cytoplasmic types, *orf79* alleles and *atp6-orf79*-like structures in wild Or-CT0 populations.
