# Supplementary material for: Multicentric origin and diversification of atp6‐orf79‐like structures reveal mitochondrial gene flows in Oryza rufipogon and Oryza sativa
Source: Evol Appl. 2020 Jun 27;13(9):2284–99. doi: 10.1111/eva.13022 (PMC7513716; doi:10.1111/eva.13022)
Supplement: Supplementary file 3 — Table S2‐S8 [file EVA-13-2284-s003.docx]

**Supplement tables**

**Table S1** The assembly statistics of mitochondrial genomes of the 590 *O. rufipogon* and *O. sativa* accessions. (Supplement data)

**Table S2** The 92 rice strains containing candidate CMS related genes.

**Table S3** Overall assembly statistics for draft mitochondrial genomes of 590 tested materials.

**Table S4** Haplotypes of *atp6*-*orf79*-like structures detected in common rice and common wild rice.

**Table S5** Geographic distribution of haplotypes of *atp6*-*orf79*-like structures in common wild rice.

**Table S6** Geographic distribution of the *orf79* alleles in common wild rice.

**Table S7** Nucleotide variations of completed sequences of 28 protein coding genes in mitochondrial genomes of 590 accessions**.**

**Table S8** The positively selective codon sites identified in different orf79 alleles based on Naive Empirical Bayes (NEB) analysis and Bayes Empirical Bayes (BEB) analysis.

**Table S2** The 92 rice accessions containing candidate CMS related genes.

| **ID** | **Accessions/Cultivars** | **Species** | **Germplasm types** | **atp6-orf79 haplotype** | **orf79 haplotype** | **Group of haplotypes** | **Candidate cytoplasm type of CMS** |
| --- | --- | --- | --- | --- | --- | --- | --- |
| IRIS_313-8655 | TAK | *O. sativa* | Aus | H1 | orf79a(BT) | AO-I | BT-CMS |
| W1080 | W1080 | *O. rufipogon* | common wild rice | H1 | orf79a(BT) | AO-I | BT-CMS |
| W1679 | W1679 | *O. rufipogon* | common wild rice | H1 | orf79a(BT) | AO-I | BT-CMS |
| W1748 | W1748 | *O. rufipogon* | common wild rice | H1 | orf79a(BT) | AO-I | BT-CMS |
| W1819 | W1819 | *O. rufipogon* | common wild rice | H1 | orf79a(BT) | AO-I | BT-CMS |
| W1865 | W1865 | *O. rufipogon* | common wild rice | H1 | orf79a(BT) | AO-I | BT-CMS |
| W1702 | W1702 | *O. bathii* | wild rice | H1 | orf79a(BT) | AO-I | BT-CMS |
| CX143 | Khasar | *O. sativa* | Aromatic | H2 | orf79a(BT) | AO-I | BT-CMS-like |
| IRIS_313-10102 | KELIRENY | *O. sativa* | Indica | H2 | orf79a(BT) | AO-I | BT-CMS-like |
| IRIS_313-11023 | BASMATI SURKH 161 | *O. sativa* | Aromatic | H2 | orf79a(BT) | AO-I | BT-CMS-like |
| IRIS_313-11218 | BADSHABHOG 4-60 | *O. sativa* | Aromatic | H2 | orf79a(BT) | AO-I | BT-CMS-like |
| IRIS_313-11411 | DONRADAO | *O. sativa* | Indica | H2 | orf79a(BT) | AO-I | BT-CMS-like |
| IRIS_313-11625 | CHOBO | *O. sativa* | Aromatic | H2 | orf79a(BT) | AO-I | BT-CMS-like |
| IRIS_313-11650 | NCS901 A | *O. sativa* | Temperate japonica | H2 | orf79a(BT) | AO-I | BT-CMS-like |
| IRIS_313-11765 | P 335 | *O. sativa* | Aus | H2 | orf79a(BT) | AO-I | BT-CMS-like |
| IRIS_313-8184 | FRAGRANCE | *O. sativa* | Indica | H2 | orf79a(BT) | AO-I | BT-CMS-like |
| IRIS_313-8288 | AVO | *O. sativa* | Indica | H2 | orf79a(BT) | AO-I | BT-CMS-like |
| IRIS_313-8326 | JC1 | *O. sativa* | Aromatic | H2 | orf79a(BT) | AO-I | BT-CMS-like |
| IRIS_313-8398 | KHARSU 80 | *O. sativa* | Aus | H2 | orf79a(BT) | AO-I | BT-CMS-like |
| IRIS_313-8614 | RAJHUSAI(ACR12) | *O. sativa* | Indica | H2 | orf79a(BT) | AO-I | BT-CMS-like |
| IRIS_313-8765 | TIMMURAY | *O. sativa* | Aromatic | H2 | orf79a(BT) | AO-I | BT-CMS-like |
| IRIS_313-8813 | ARC 10497 | *O. sativa* | Aromatic | H2 | orf79a(BT) | AO-I | BT-CMS-like |
| IRIS_313-8814 | SHANKA | *O. sativa* | Aromatic | H2 | orf79a(BT) | AO-I | BT-CMS-like |
| IRIS_313-9039 | MUTTU SAMBA | *O. sativa* | Indica | H2 | orf79a(BT) | AO-I | BT-CMS-like |
| IRIS_313-9629 | JC157 | *O. sativa* | Aus | H2 | orf79a(BT) | AO-I | BT-CMS-like |
| IRIS_313-9778 | GENIT | *O. sativa* | Indica | H2 | orf79a(BT) | AO-I | BT-CMS-like |
| IRIS_313-9978 | IRAT 118 | *O. sativa* | Temperate japonica | H2 | orf79a(BT) | AO-I | BT-CMS-like |
| W0128 | W0128 | *O. rufipogon* | common wild rice | H2 | orf79a(BT) | AO-I | BT-CMS-like |
| W0589 | W0589 | *O. rufipogon* | common wild rice | H3 | orf79a(BT) | AO-I | BT-CMS-like |
| W1111 | W1111 | *O. rufipogon* | common wild rice | H5 | orf79a(BT) | AO-I | BT-CMS-like |
| IRIS_313-11458 | KARAHANI | *O. sativa* | Temperate japonica | H6 | orf79a(BT) | AO-I | BT-CMS-like |
| IRIS_313-12074 | KYAIN WAR ME DONE | *O. sativa* | Aromatic | H6 | orf79a(BT) | AO-I | BT-CMS-like |
| IRIS_313-8252 | JAGLI BORO | *O. sativa* | Aus | H6 | orf79a(BT) | AO-I | BT-CMS-like |
| IRIS_313-8321 | BHADOIA 303 | *O. sativa* | Aus | H6 | orf79a(BT) | AO-I | BT-CMS-like |
| W1084 | W1084 | *O. rufipogon* | common wild rice | H6 | orf79a(BT) | AO-I | BT-CMS-like |
| W1086 | W1086 | *O. rufipogon* | common wild rice | H6 | orf79a(BT) | AO-I | BT-CMS-like |
| W1092 | W1092 | *O. rufipogon* | common wild rice | H6 | orf79a(BT) | AO-I | BT-CMS-like |
| W1747 | W1747 | *O. rufipogon* | common wild rice | H6 | orf79a(BT) | AO-I | BT-CMS-like |
| IRIS_313-11191 | RANRUWAN | *O. sativa* | Aus | H7 | orf79a(BT) | AO-I | BT-CMS-like |
| W1881 | W1881 | *O. rufipogon* | common wild rice | H11 | orf79k(HL) | AO-III | HL-CMS |
| W3002 | W3002 | *O. rufipogon* | common wild rice | H11 | orf79k(HL) | AO-III | HL-CMS |
| W3005 | W3005 | *O. rufipogon* | common wild rice | H11 | orf79k(HL) | AO-III | HL-CMS |
| W3057 | W3057 | *O. rufipogon* | common wild rice | H11 | orf79k(HL) | AO-III | HL-CMS |
| W3061 | W3061 | *O. rufipogon* | common wild rice | H11 | orf79k(HL) | AO-III | HL-CMS |
| W3066 | W3066 | *O. rufipogon* | common wild rice | H11 | orf79k(HL) | AO-III | HL-CMS |
| W0132 | W0132 | *O. rufipogon* | common wild rice | H12 | orf79k(HL) | AO-III | HL-CMS-like |
| W1766 | W1766 | *O. rufipogon* | common wild rice | H12 | orf79k(HL) | AO-III | HL-CMS-like |
| W1780 | W1780 | *O. rufipogon* | common wild rice | H12 | orf79k(HL) | AO-III | HL-CMS-like |
| W0157 | W0157 | *O. rufipogon* | common wild rice | H13 | orf79k(HL) | AO-III | HL-CMS-like |
| W1119 | W1119 | *O. rufipogon* | common wild rice | H13 | orf79k(HL) | AO-III | HL-CMS-like |
| W1124 | W1124 | *O. rufipogon* | common wild rice | H13 | orf79k(HL) | AO-III | HL-CMS-like |
| B192 | R42 | *O. sativa* | Intermediate | H4 | orf79b(LD) | AO-I | LD-CMS |
| IRIS_313-11657 | EX WUKARI(WILD) | *O. sativa* | Tropical japonica | H4 | orf79b(LD) | AO-I | LD-CMS |
| IRIS_313-7725 | MADINIKA 1329 | *O. sativa* | Temperate japonica | H4 | orf79b(LD) | AO-I | LD-CMS |
| IRIS_313-9551 | BENGALY MORIMO | *O. sativa* | Indica | H4 | orf79b(LD) | AO-I | LD-CMS |
| W1683 | W1683 | *O. rufipogon* | common wild rice | H4 | orf79b(LD) | AO-I | LD-CMS |
| W3069 | W3069 | *O. rufipogon* | common wild rice | H10 | orf79j | AO-III | novel type |
| W3070 | W3070 | *O. rufipogon* | common wild rice | H10 | orf79j | AO-III | novel type |
| W0153 | W0153 | *O. rufipogon* | common wild rice | H14 | orf79g | AO-II | novel type |
| W0549 | W0549 | *O. rufipogon* | common wild rice | H14 | orf79g | AO-II | novel type |
| W1809 | W1809 | *O. rufipogon* | common wild rice | H14 | orf79g | AO-II | novel type |
| W0171 | W0171 | *O. rufipogon* | common wild rice | H15 | orf79i | AO-II | novel type |
| W1715 | W1715 | *O. rufipogon* | common wild rice | H16 | orf79e | AO-IV | novel type |
| W1716 | W1716 | *O. rufipogon* | common wild rice | H16 | orf79e | AO-IV | novel type |
| W1724 | W1724 | *O. rufipogon* | common wild rice | H16 | orf79e | AO-IV | novel type |
| W1725 | W1725 | *O. rufipogon* | common wild rice | H16 | orf79e | AO-IV | novel type |
| W3013 | W3013 | *O. rufipogon* | common wild rice | H16 | orf79e | AO-IV | novel type |
| W3020 | W3020 | *O. rufipogon* | common wild rice | H16 | orf79e | AO-IV | novel type |
| W3021 | W3021 | *O. rufipogon* | common wild rice | H16 | orf79e | AO-IV | novel type |
| W3022 | W3022 | *O. rufipogon* | common wild rice | H16 | orf79e | AO-IV | novel type |
| W3023 | W3023 | *O. rufipogon* | common wild rice | H16 | orf79e | AO-IV | novel type |
| W3034 | W3034 | *O. rufipogon* | common wild rice | H16 | orf79e | AO-IV | novel type |
| W3009 | W3009 | *O. rufipogon* | common wild rice | H8 | orf79c | AO-I | novel type |
| W3085 | W3085 | *O. rufipogon* | common wild rice | H9 | orf79f | AO-I | novel type |
| W3086 | W3086 | *O. rufipogon* | common wild rice | H9 | orf79f | AO-I | novel type |
| IRIS_313-11026 | HANSRAJ | *O. sativa* | Aromatic | - | orf79a(BT) | - | unknown |
| IRIS_313-8647 | PERUNEL | *O. sativa* | Indica | - | orf79a(BT) | - | unknown |
| W0168 | W0168 | *O. rufipogon* | common wild rice | - | orf79e | - | unknown |
| W0574 | W0574 | *O. rufipogon* | common wild rice | - | orf79a(BT) | - | unknown |
| W1082 | W1082 | *O. rufipogon* | common wild rice | - | orf79a(BT) | - | unknown |
| W1083 | W1083 | *O. rufipogon* | common wild rice | - | orf79a(BT) | - | unknown |
| W1735 | W1735 | *O. rufipogon* | common wild rice | - | orf79a(BT) | - | unknown |
| W1740 | W1740 | *O. rufipogon* | common wild rice | - | orf79a(BT) | - | unknown |
| W1741 | W1741 | *O. rufipogon* | common wild rice | - | orf79a(BT) | - | unknown |
| W1742 | W1742 | *O. rufipogon* | common wild rice | - | orf79d | - | unknown |
| W1746 | W1746 | *O. rufipogon* | common wild rice | - | orf79a(BT) | - | unknown |
| W1757 | W1757 | *O. rufipogon* | common wild rice | - | orf79a(BT) | NA3-orf79 | unknown |
| W1759 | W1759 | *O. rufipogon* | common wild rice | - | orf79a(BT) | - | unknown |
| W1777 | W1777 | *O. rufipogon* | common wild rice | - | orf79a(BT) | - | unknown |
| W1782 | W1782 | *O. rufipogon* | common wild rice | - | orf79d | NA1-orf79 | unknown |
| W3071 | W3071 | *O. rufipogon* | common wild rice | - | orf79h | NA2-orf79 | unknown |
| W3094 | W3094 | *O. rufipogon* | common wild rice | - | orf79k(HL) | - | unknown |

**Table S3** Overall assembly statistics for draft mitochondrial genomes of 590 tested materials.

| Subject | Average | Max | Min | STD |
| --- | --- | --- | --- | --- |
| Original WGS dataset |  |  |  |  |
| Total number of reads | 4637567.64 | 11919951 | 2066553 | 2273930.06 |
| Total length of reads | 947581357 | 2789268534 | 380334220 | 497117924.4 |
| Estimated average coverage depth | 2.53 | 7.45 | 1.02 | - |
| Mitochondrial genome assemblies | | | | |
| Filtered reads | 532662.36 | 2555310.00 | 124920.00 | 280773.40 |
| Mapped reads (%) | 98.08 | 99.17 | 93.57 | 0.63 |
| Avg. coverage depth | 111.56 | 669.00 | 29.00 | 61.72 |
| Contigs (≥150 bp) | 112.85 | 566.00 | 47.00 | 70.62 |
| Contigs (≥1000 bp) | 36.56 | 113.00 | 24.00 | 7.79 |
| Contigs (≥5000 bp) | 15.06 | 29.00 | 9.00 | 3.58 |
| Contigs (≥ 10000 bp) | 10.51 | 16.00 | 6.00 | 2.13 |
| Contigs (≥ 25000 bp) | 4.95 | 8.00 | 0.00 | 1.28 |
| Contigs (≥50000 bp) | 1.71 | 4.00 | 0.00 | 1.10 |
| Total length (≥ 150 bp) | 419999.90 | 600250.00 | 389749.00 | 24863.16 |
| Total length (≥1000 bp) | 390865.33 | 513470.00 | 356056.00 | 10729.04 |
| Total length (≥5000 bp) | 348644.61 | 387661.00 | 259049.00 | 15306.34 |
| Total length (≥ 10000 bp) | 316862.36 | 363966.00 | 155297.00 | 26512.62 |
| Total length (≥25000 bp) | 229352.83 | 329962.00 | 0.00 | 63365.15 |
| Total length (≥50000 bp) | 117187.12 | 279800.00 | 0.00 | 75170.74 |
| Largest contig (bp) | 72960.71 | 149503.00 | 20409.00 | 27106.48 |
| GC (%) | 43.34 | 44.03 | 41.75 | 0.23 |
| N50 | 33665.19 | 89120.00 | 7111.00 | 15241.20 |
| NG50 | 25092.56 | 56477.00 | 5724.00 | 9318.98 |

**Table S4** Haplotypes of *atp6*-*orf79*-like structures detected in Asia cultivated rice and common wild rice.

| Haplotypes | Total | Asia cultivated rice | | | | | | Common wild rice |
| --- | --- | --- | --- | --- | --- | --- | --- | --- |
|  |  | Total | Japonica | intermediate | Indica | Aus | Aromtic |  |
| H1 | 6 | 1 |  |  |  | 1(4.35%) |  | 5 |
| H2 | 21 | 20 | 2(1.13%) |  | 7(5.51%) | 3(13.04%) | 8(33.33%) | 1 |
| H3 | 1 | 0 |  |  |  |  |  | 1 |
| H4 | 5 | 4 | 2(1.13%) | 1(5.26%) | 1(0.79%) |  |  | 1 |
| H5 | 1 | 0 |  |  |  |  |  | 1 |
| H6 | 8 | 4 | 1(0.57%) |  |  | 2(8.70%) | 1(4.17%) | 4 |
| H7 | 1 | 1 |  |  |  | 1(4.35%) |  | 0 |
| H8 | 1 | 0 |  |  |  |  |  | 1 |
| H9 | 2 | 0 |  |  |  |  |  | 2 |
| H10 | 2 | 0 |  |  |  |  |  | 2 |
| H11 | 6 | 0 |  |  |  |  |  | 6 |
| H12 | 3 | 0 |  |  |  |  |  | 3 |
| H13 | 3 | 0 |  |  |  |  |  | 3 |
| H14 | 3 | 0 |  |  |  |  |  | 3 |
| H15 | 1 | 0 |  |  |  |  |  | 1 |
| H16 | 10 | 0 |  |  |  |  |  | 10 |
|  |  |  |  |  |  |  |  |  |
| AO-I | 46 | 30 | 5(2.84%) | 1(5.26%) | 8(6.30%) | 7(30.43%) | 9(37.50%) | 16 |
| AO-III | 14 | 0 | 0 | 0 | 0 | 0 | 0 | 14 |
| AO-II | 4 | 0 | 0 | 0 | 0 | 0 | 0 | 4 |
| AO-IV | 10 | 0 | 0 | 0 | 0 | 0 | 0 | 10 |
|  |  |  |  |  |  |  |  |  |
| Total haplotypes | 74 | 30 | 5 | 1 | 8 | 7 | 9 | 44 |
| Total accessions | 590 | 369 | 176 | 19 | 127 | 23 | 24 | 221 |
| Total Frequency | 0.13 | 0.08 | 0.03 | 0.05 | 0.06 | 0.30 | 0.38 | 0.20 |

**Table S5** Geographic distribution of haplotypes of *atp6*-*orf79*-like structures in common wild rice.

| Haplotype | Total number | Pairwise geographic distance (km) | | | GCF^1^ | | | GMC^2^ | | |
| --- | --- | --- | --- | --- | --- | --- | --- | --- | --- | --- |
|  |  | Average | SE | Range | coordinate | location | coordinate^1^ | | location |  |
| H1 | 5 | 1388.38 | 730.01 | 417.86-2946.53 | 23.71, 90.41 | SA-I | 23.66, 89.12 | | SA-I |  |
| H2 | 1 | - | - | - | 21.0, 85.1 | SA-II | 21, 85.1 | | SA-II |  |
| H3 | 1 | - | - | - | 5.7, 102.53 | SEA | 5.7, 102.53 | | SEA |  |
| H4 | 1 | - | - | - | 20.1, 84.48 | SA-II | 20.1, 84.48 | | SA-II |  |
| H5 | 1 | - | - | - | 26.82, 94.17 | SA-I | 26.82, 94.17 | | SA-I |  |
| H6 | 4 | 794.72 | 643.11 | 0-1585.02 | 27.0, 88.4 | SA-I | 26.95, 88.42 | | SA-I |  |
| H8 | 1 | - | - | - | 22.89, 112.85 | EA | 22.89, 112.85 | | EA |  |
| H9 | 2 | 0 | - | 0 | 23.6, 102.01 | EA | 23.6, 102.01 | | EA |  |
| H10 | 2 | 0 | - | 0 | 18.65, 109.8 | EA | 18.65, 109.8 | | EA |  |
| H11 | 6 | 509.93 | 359.64 | 0-1185.82 | 19.5, 109.5 | EA | 19.43, 109.51 | | EA |  |
| H12 | 3 | 328.09 | 248.82 | 40.78-472.92 | 17.0, 81.8 | SA-II | 17.1, 82.03 | | SA-II |  |
| H13 | 3 | 17.46 | 14.26 | 0.99-25.75 | 26.59, 94.19 | SA-I | 26.59, 94.17 | | SA-I |  |
| H14 | 3 | 1416.04 | 631.40 | 722.31-1957.13 | 13.0, 77.6 | SA-II | 12.83, 78.3 | | SA-II |  |
| H15 | 1 | - | - | - | 15.87, 100.99 | SEA | 15.87, 100.99 | | SEA |  |
| H16 | 10 | 574.39 | 688.82 | 0-1725.99 | 23.17, 112.89 | EA | 23.4, 112.9 | | EA |  |

^1^Geographic central feature (GCF) was defined as the genotype that has the shortest average distance with other genotypes.

^2^ Geographic Median Center (GMC) was defined as a theoretical coordinate that has the shortest average distance with other genotypes.

**Table S6** Geographic distribution of the *orf79* alleles in common wild rice.

| Haplotype | Total number | Pairwise geographic distance (km) | | | GCF^1^ | | | GMC^2^ | |
| --- | --- | --- | --- | --- | --- | --- | --- | --- | --- |
|  |  | Average | SE | Range | coordinate | location | coordinate | | location |
| *orf79a* | 22 | 1349.7 | 993.54 | 0-3805.2 | 27.0, 88.4 | SA-I | | 24.52, 86.03 | SA-I |
| *orf79b* | 1 | - | - | - | 20.1, 84.48 | SA-II | | 20.1, 84.48 | SA-II |
| *orf79c* | 1 | - | - | - | 22.89, 112.85 | EA | | 22.89, 112.85 | EA |
| *orf79d* | 2 | 1626.8 | 0 | 1626.8 | - | - | | 19.615, 76.23 | SA-II |
| *orf79e* | 8 | 738.6 | 692.11 | 0-1726.0 | 23.17, 112.89 | EA | | 23.22, 112.87 | EA |
| *orf79f* | 2 | 0 | 0 | 0 | 23.6, 102.01 | EA | | 23.6, 102.01 | EA |
| *orf79g* | 3 | 1416 | 631.4 | 722.3-1957.1 | 13.0, 77.6 | SA-II | | 12.83, 78.29 | SA-II |
| *orf79h* | 1 | - | - | - | 19.62, 110.7 | EA | | 19.62, 110.7 | EA |
| *orf79i* | 1 | - | - | - | 15.87, 100.99 | SEA | | 15.87, 100.99 | SEA |
| *orf79j* | 2 | 0 | 0 | 0 | 18.65, 109.8 | EA | | 18.65, 109.8 | EA |
| *orf79k* | 13 | 1606.4 | 1006.43 | 0-3291.8 | 19.5, 109.5 | EA | | 21.42, 103.51 | EA |

^1^Geographic central feature (GCF) was defined as the genotype that has the shortest average distance with other genotypes.

^2^ Geographic Median Center (GMC) was defined as a theoretical coordinate that has the shortest average distance with other genotypes.

**Table S7** Nucleotide variations of completed sequences of 28 protein coding genes detected in mitochondrial genomes of 590 accessions.

| Gene | Number of alleles | Total length | Number of Variations | | |
| --- | --- | --- | --- | --- | --- |
|  |  |  | Total | Indel | SNP |
| *orf79* | 11 | 240 | 9 | 0 | 9 |
| *cox3* | 5 | 843 | 4 | 1 | 3 |
| *cox2* | 8 | 2049, 2058, 2061, 2063 | 12 | 3 | 9 |
| *rps1* | 13 | 512, 519 | 15 | 3 | 12 |
| *rps4* | 3 | 1059 | 3 | 0 | 3 |
| *orf162* | 2 | 489 | 2 | 0 | 2 |
| *orf176* | 2 | 531 | 1 | 1 | 0 |
| *orf181* | 2 | 546 | 1 | 1 | 0 |
| *atp1* | 1 | 1530 | 0 | 0 | 0 |
| *atp6* | 1 | 1005 | 0 | 0 | 0 |
| *atp9* | 1 | 225 | 0 | 0 | 0 |
| *ccmB* | 1 | 621 | 0 | 0 | 0 |
| *ccmC* | 1 | 723 | 0 | 0 | 0 |
| *ccmFn* | 1 | 1785 | 0 | 0 | 0 |
| *nad3* | 1 | 468 | 0 | 0 | 0 |
| *nad6* | 1 | 618 | 0 | 0 | 0 |
| *nad9* | 1 | 573 | 0 | 0 | 0 |
| *orf153* | 1 | 462 | 0 | 0 | 0 |
| *orf161* | 1 | 486 | 0 | 0 | 0 |
| *orf25* | 1 | 594 | 0 | 0 | 0 |
| *orf258* | 1 | 778 | 0 | 0 | 0 |
| *orf490* | 1 | 1473 | 0 | 0 | 0 |
| *orfX* | 1 | 811 | 0 | 0 | 0 |
| *rpl5* | 1 | 567 | 0 | 0 | 0 |
| *rps13* | 1 | 351 | 0 | 0 | 0 |
| *rps7* | 1 | 447 | 0 | 0 | 0 |
| *rps12* | 1 | 378 | 0 | 0 | 0 |
| *rps19* | 1 | 282 | 0 | 0 | 0 |

**Table S8** The positively selective codon sites identified in different *orf79* alleles based on Naive Empirical Bayes (NEB) analysis and Bayes Empirical Bayes (BEB) analysis

| No. | Nuc. variation | AA  variation | Pr_NEB_ (ω>1)^a^ | ω_NEB_  (mean±SE) | Pr_BEB_  (ω>1) | ω_BEB_  (mean±SE) |
| --- | --- | --- | --- | --- | --- | --- |
| scs1 | 4 g/a | 2 A/T | 1.000**^b^ | 64.928±0 | 0.987* | 9.115±1.722 |
| scs2 | 13 g/c | 5 V/L | 1.000** | 64.928±0 | 0.854 | 7.962±3.297 |
| scs3 | 142 a/t/c/g | 48 M/L/V | 1.000** | 64.928±0 | 1.000** | 9.220±1.449 |
| scs4 | 146 a/c  147 a/c/t | 49 E/D/A | 1.000** | 64.928±0 | 0.986* | 9.106±1.742 |
| scs5 | 178 c/t | 60 H/Y | 1.000** | 64.928±0 | 0.986* | 9.107±1.739 |

^a^ω indicates the dN/dS.

^b^*indicates probability of Pr > 95% while ** indicates Pr > 99%.
